# Establishment of a laboratory mouse model to study *Borrelia miyamotoi* infection and disease

**DOI:** 10.3389/fimmu.2026.1851617

**Published:** 2026-06-12

**Authors:** Linda K. Bockenstedt, Alexia A. Belperron, Jialing Mao, Caroline J. Zeiss, Alan G. Barbour, Michel Ledizet, Peter J. Krause, Kenneth R. Dardick

**Affiliations:** 1 Dept of Internal Medicine, Section of Rheumatology, Allergy and Immunology, Yale School of Medicine, New Haven, CT, United States; 2 Department of Comparative Medicine, Yale School of Medicine, New Haven, CT, United States; 3 Departments of Medicine and Microbiology & Molecular Genetics, University of California, Irvine, Irvine, CA, United States; 4 L2 Diagnostics, LLC, New Haven, CT, United States; 5 Department of Epidemiology of Microbial Diseases, Yale School of Public Health, Department of Internal Medicine, Department of Pediatrics, Yale School of Medicine, New Haven, CT, United States; 6 Mansfield Family Practice, Storrs, CT, United States

**Keywords:** *Borrelia miyamotoi*, *Ixodes ticks*, mouse, relapsing fever, spirochetes

## Abstract

*Borrelia miyamotoi* is an emerging relapsing fever spirochete transmitted by *Ixodes* spp. ticks that serve as vectors for other human pathogens, including the agents of Lyme disease. To date, an animal model to study tick-borne *B. miyamotoi* infection and pathology has not been reported. Here, we describe the development of a laboratory mouse model of infection using clinical isolate CT14D4 derived from the blood of a Connecticut resident with disseminated Lyme disease. Genomic analysis revealed that the CT14D4 genome was similar to other *B. miyamotoi* strains isolated from *Ixodes scapularis* ticks in the eastern United States. We show that CT14D4 can be propagated in mice by needle inoculation and through a tick–mouse infection cycle. Relapsing spirochetemia was observed in wild-type (WT), *Myd88*^−/−^, and splenectomized mice, all of which eventually cleared the infections. In contrast, *Rag1*^−/−^ mice lacking B and T cells remain persistently bacteremic. WT mice that had cleared CT14D4 infection are resistant to reinfection with this *B. miyamotoi* strain. Spirochetes were visualized in several organs after perfusion fixation of infected *Rag1*^−/−^ mice, including the brain, with histopathology revealing extramedullary hematopoiesis and inflammatory infiltrates that were most pronounced in the liver. WT mice exhibited similar but milder pathology during periods of bacteremia. These studies provide a useful model to study hard-tick relapsing fever pathogenesis and disease.

## Introduction

Relapsing fever (RF) infections occur worldwide and are caused by several spirochetes of the genus *Borrelia* (1, 2). Most RF spirochetes are transmitted by fast-feeding soft-bodied ticks, whereas a single species (*Borrelia recurrentis*) is transmitted by lice (2). In 1995, a new RF genospecies *Borrelia miyamotoi* was identified in hard-bodied *Ixodes* ticks that serve as vectors for pathogens causing other human diseases, including Lyme disease, babesiosis, anaplasmosis, and tick-borne encephalitis (3). Since then, *B. miyamotoi* has been identified in ticks throughout the northern hemisphere (4). Genomic analyses have revealed that *B. miyamotoi* strains cluster geographically by *Ixodes* genospecies with little diversity exhibited among isolates within a tick species (5). In endemic areas, the prevalence of *B. miyamotoi* in *Ixodes* ticks can be as high as two-thirds that found for *B. burgdorferi sensu lato* spirochetes that cause Lyme disease (6–13).

The first human cases of *B. miyamotoi* disease (BMD) were described in Russia in 2011 and have now been reported in North America, Europe, and Asia where *B. miyamotoi*-infected *Ixodes* ticks have been found (14–19). Case finding studies of human infections report signs and symptoms ranging from mild viral-like illnesses to meningoencephalitis with cognitive and motor dysfunction (14–16, 19–24). In the United States, seroprevalence studies have found exposure rates to *B. miyamotoi* as high as 1%–5% in endemic areas (11, 15), suggesting that asymptomatic infection may be occurring and/or that milder illnesses may go undiagnosed. BMD is more severe in immunocompromised individuals and most often has been associated with the use of B cell-depleting agents (18, 19, 21, 24). The spectrum of BMD infection and host factors that increase susceptibility to more severe disease, however, are not fully known. While various *B. miyamotoi* isolates have been shown to infect mice, an animal model to investigate BMD pathogenesis has not yet been reported.

In this study, we describe the development and characterization of a laboratory mouse model for BMD using a *B. miyamotoi* clinical isolate derived from the blood of a Connecticut resident presenting with disseminated Lyme disease. Initial genomic sequencing of this isolate, named CT14D4, revealed nearly complete identity with *B. miyamotoi* strain LB-2001 previously obtained from xenodiagnostic ticks that fed on a *Peromyscus leucopus* mouse that had been trapped in Connecticut (3). Our results using multiple routes of infection demonstrate that CT14D4 can cause relapsing spirochetemia in immunocompetent inbred mouse strains. Although bacteremia resolves without evidence of ongoing infection in immunocompetent and certain immune-deficient mouse strains, *Rag1*^−/−^ mice that lack T and B cells exhibit persistent spirochetemia that can result in death. Unlike other *B. miyamotoi* isolates studied in mice, CT14D4 can be acquired from and transmit infection to *Rag1*^−/−^ and wild-type (WT) mice by tick bite, is visualized in blood samples, and can be detected in selected tissues of infected *Rag1*^−/−^ mice after perfusion fixation, including the brain. Histopathology of infected tissues demonstrates extramedullary hematopoiesis and prominent hepatic inflammation that is more severe in *Rag1*^−/−^ mice than in WT mice. Other organ systems can be affected. The establishment of a reproducible infection model and the pathology associated with *B. miyamotoi* infection has provided a more in-depth understanding of immunopathogenesis of this emerging human pathogen and highlights differences between *B. miyamotoi* infection and RF due to pathogens transmitted by soft ticks and lice.

## Methods

### Mice

The following mice were purchased from Jackson Laboratories: C57BL/6 (B6) WT mice, splenectomized B6 mice, B6;129P2-Fcer1*^gtm1Rav^*/J (FcϵRγ^−/−^) mice, and DBA-1J mice, all 6–8 weeks of age. B6.129S7-*Rag1^tm1Mom^*/J (B6 *Rag1*^−/−^) breeding pairs were a kind gift of Ruslan Medzhitov (Yale University). WT B6 mice (age 4–6 weeks) were purchased from the Charles River Laboratory for one experiment. Colonies of *Myd88*^–/–^ mice on the B6 and C3H/HeN backgrounds had been established previously in the Bockenstedt lab (25, 26). WT B6, B6 *Myd88*^−/−^, C3H *Myd88*^−/−^, and B6 *Rag1*^−/−^ mice were maintained as colonies in specific pathogen-free housing with autoclaved food, water, and bedding. Sulfamethoxazole-trimethoprim (0.25 mg/mL; Sulfatrim, Sigma-Aldrich) was added to drinking water of the *Myd88*^−/−^ mice to reduce opportunistic infection. Sulfatrim has no effect on *B. miyamotoi* infection and was provided to *Myd88*^−/−^ mice throughout all experiments. In one experiment, WT and *Myd88*^−/−^ mice that had resolved bacteremia were immunosuppressed with cortisone (3 mg/mouse/day) administered intraperitonally (i.p.) daily for 5 days immediately prior to euthanasia (27). Mice were housed in filter frame cages and administered food and water *ad libitum* according to Yale University animal care and use guidelines. All mice were euthanized by carbon dioxide (CO_2_) asphyxiation using a compressed CO_2_ cylinder with a flow regulator set to achieve a displacement of 30%–70% of the cage volume/min, according to the American Veterinary Medical Association Guidelines for the euthanasia of animals, 2020 edition (https://www.avma.org/resources-tools/avma-policies/avma-guidelines-euthanasia-animals). The Yale University Institutional Animal Care and Use Committee approved all procedures.

### Isolation of CT14D4

The clinical isolate of *B. miyamotoi* CT14D4 was derived from the blood of a Connecticut resident with multiple erythema migrans (**Supplementary Figure 1A**) who had been enrolled in an ongoing study of Lyme and other tickborne diseases approved by the Yale University Human Investigation Committee. As part of that study, blood samples were obtained to screen for potential coinfections with other *Ixodes* tick-transmitted pathogens, including *B. miyamotoi* and *Babesia microti*. Citrate-buffered blood (100 μL) from the subject was inoculated i.p. into a B6 *Rag1*^−/−^ mouse. Fourteen days after inoculation, a Giemsa-stained blood sample revealed only spirochetes and no red blood cell inclusions consistent with *Babesia* (**Supplementary Figure 1B**). This mouse was subsequently used as a source of blood or plasma containing spirochetes for mouse infections, cryopreservation of infected mouse blood or plasma in 14% glycerol, and as a blood meal host for laboratory-reared *Ixodes* ticks. All procedures were approved by the Yale University Human Investigation Committee and the Yale University Animal Care and Use Committee.

### CT14D4 genome sequencing

Cryopreserved infected mouse plasma obtained from the primary B6 *Rag1*^−/−^ mouse was thawed and inoculated i.p. into SCID mice (C.BKa-Ighb/IcrCrl; Charles River) under a University of California, Irvine approved animal care and use protocol as previously described (28). When bacterial densities in the blood reached ~10^7^ cells/mL, the mice were terminally exsanguinated under anesthesia. Total DNA was extracted from whole blood with the Qiagen DNeasy blood and tissue kit (Valencia, CA) and treated with RNase I. Multiplex quantitative PCR that discriminated between *B. burgdorferi* and *B. miyamotoi* was carried out as previously described with negative and positive controls (29). For next-generation sequencing, libraries were produced with the Ion Xpress Plus fragment library kit with size selection by electrophoresis before emulsion PCR on an Ion OneTouch apparatus (Life Technologies, Carlsbad, CA). Sequencing was carried out on an Ion Torrent PGM apparatus with 200-bp nucleotide chemistry and four Ion 316 Chips (Life Technologies). Reads of 75 to 245 nucleotides (nt) were filtered with the 2.6-Gb *Mus musculus* sequence of Genome Reference Consortium Mouse Build 38 (assembly average length of 193 nt). With the Assembly Cell algorithm of the CLC Genomics Workbench v. 7.5.1 (CLC bio, Denmark), these were assembled *de novo* into 35 DNA contigs with an average length 29,068 bp and average coverage of 1944×. With the BMSL LB-2001 chromosome (GenBank CP006647) as the reference, 6,417,381 reads were mapped for a single contig of 907,293 nts with these settings. The *de novo* assembled contigs were matched with the mapped-to-reference sequence for error detection and correction. GenBank accession number CP010308 has been assigned to the chromosome of CT14D4. The ~900-kb linear chromosome sequences of CT14D4 and *B. miyamotoi* strains LB-2001 from Connecticut (CP006647), CT13–2396 from Connecticut (CP017126), MN18–0001 from Minnesota (CP150054), CA17–2241 from California (CP021872), and HT31 from Japan (AP024371) were aligned with MAFFT v. 7 (https://mafft.cbrc.jp). Nucleotide polymorphisms were analyzed with DnaSP v. 6 (https://www.ub.edu/dnasp/).

### Enumeration of CT14D4 in blood samples

Blood samples were collected either by retroorbital bleeding or by tail vein bleeds depending on the volume extracted or frequency of sampling. Blood samples (100 µL) were collected by retroorbital bleeding and mixed 1:1 with Alsever’s solution (Sigma-Aldrich) in Eppendorf tubes. Blood cells were pelleted at 1,200 rpm for 3 min, after which 8 μL of the plasma supernatant was applied to individual wells of a 12-well glass slide under a cover slip and examined by darkfield (DF) microscopy for the presence of spirochetes. To enumerate the total spirochetes in the sample, spirochetes in the remaining supernatant were pelleted at 8,000 rpm, then resuspended in 5 µL of residual supernatant. The 5-µL volume was then applied onto a 12-well glass slide and spirochetes were counted in 30 visual fields in each well. When pathogen burdens were high, samples were diluted to permit more accurate counting. When multiple blood draws were obtained from the same mouse, 10-µL blood samples were obtained from the tail veins and mixed 1:1 with Alsever’s solution. Blood cells were pelleted at 1,200 rpm for 3 min, and spirochetes were enumerated in the indicated amount of the residual supernatant as described above. Alternatively, pathogen burdens were enumerated in plasma smears that were air dried, fixed in cold methanol, and stained with FITC anti-*Borrelia* species antibodies (originally KPL, Inc, now SeraCare) as described below (see the *Immunofluorescent staining of spirochetes* section).

### PCR detection of *B. miyamotoi* DNA

PCR was performed using 1 μL of whole blood or plasma and the Phusion Blood Direct PCR kit (Thermo Scientific) with the *B. miyamotoi flaB* gene as the target. The reaction was carried out with Qiagen Taq Polymerase in 25 µL total volume using the following primers and reaction conditions: forward primer 5′ gcatcattagctggaacacaagc (bp366–389) and reverse primer 5′ aactggagcggctgctggagc (bp547–567), 66°C annealing temperature, 35 cycles. For plasma, 100 spirochetes/1 µL correlated with the detection of at least one spirochete by DF in three to five visual fields. The same primers and PCR reactions were used on tick DNA, which was extracted from engorged ticks using the Allprep DNA/RNA Mini Kit (QIAGEN). Quantitative PCR for DNA encoding the 16S RNA subunit of *B. miyamotoi* was performed by L2 Diagnostics using forward primer 5′ acgcgtggataatctacctac and reverse primer 5′ aagacgcagactcatctacaagc. DNA purified from 5 µL of mouse blood was eluted into 60 µL of RNase-free water. DNA solution (5 µL) was used in each reaction. A total of 40 cycles of amplification were performed, with an annealing temperature of 55.9°C. Amplification products were detected using a custom 5′ FAM-labeled, 3′ MGB-modified TaqMan probe with the sequence CCGAATAAAGTCAATTGAGGTG purchased from Thermo Fisher. For PCR of mouse tissues, DNA was isolated using the NucleoSpin Tissue Kit according to the manufacturer’s recommendations (Machery Nagel). One microliter of DNA eluted into 100 µL of BE buffer was used in each reaction. Amplification of the mouse *tubulin* gene was carried out with the following primers and reaction conditions: forward primer 5′ ggcgccctctgtgtagtggcctttggccca and reverse primer 5′ caggctggtcaatgtggcaaccagatggt, 55°C annealing temperature, 35 cycles.

### Tick infestation of mice

Laboratory-reared specific pathogen-free *Ixodes scapularis* larvae were provided by Durland Fish (Yale School of Public Health) and also purchased from the National Tick Research and Education Resource (Oklahoma State University, Stillwater, OK). Larvae were allowed to feed to repletion on uninfected or infected mice to generate nymphs as previously described (27). Mice were anesthetized prior to each tick infestation. Fed larvae were collected and maintained in environmental chambers set at 22 °C and >90% relative humidity. After molting, nymphs were stored at 8 °C and in >90% relative humidity until experimental infestations were performed as previously described (27).

### Immunofluorescent staining of spirochetes

Air-dried blood, plasma, or cerebrospinal fluid (CSF) smears were fixed in cold methanol for 5 min, rinsed with PBS, and blocked in 5% normal goat serum (Gibco) in PBS for 30 min at room temperature (RT). Samples were stained with a 1:30 dilution of FITC anti-*Borrelia* species antibodies in blocking buffer for 1 h at RT in the dark. Slides were washed and coverslips were mounted in Fluoromount-G (Southern Biotech) and sealed. Sections from formalin-fixed paraffin-embedded (FFPE) tissues were deparaffinized, subjected to antigen retrieval with 10 mM sodium citrate according to standard methods, blocked with 5% goat serum, and then stained with FITC anti-*Borrelia* species antibodies (1:30 dilution) and DAPI to identify cell nuclei. Tick midguts were dissected in phosphate-buffered saline, applied to 12-well slides, air dried, and acetone fixed prior to staining with FITC anti-*Borrelia* species antibodies. Stained smears and tissues were examined using an Olympus BX-40 fluorescent microscope with UPLanF1, 40×/0.17 and UPLanF1, 20×/0.50 objectives. Images were captured with a Spot RT3 camera using Spot software version 5.2.

### CT14D4 culture

One-milliliter aliquots of pooled frozen plasma containing 5 × 10^6^ spirochetes/mL from B6 *Rag1*^−/−^ or WT mice infected by tick bite were cultured in 7 mL of Barbour–Stoenner–Kelley (BSK) II medium in screw-top tubes at 33 °C. After 2 weeks, when the density of spirochetes reached ~10^6^/mL, as enumerated using a Petroff–Hausser counting chamber, either spirochetes were used to infect mice by needle inoculation or the culture was split 1:1 into new BSK II media in a total volume of 7 mL. For further expansion of spirochetes, cultures grown to ~10^7^ spirochetes/mL were split 1:10 into new BSK II media in a total volume of 50 mL, typically reaching the density ~10^7^/mL after 2 weeks. These large-volume cultures were used to generate cell lysates for SDS-PAGE and immunoblot.

### Immunoblot

Bacterial lysates were generated from cultured CT14D4 spirochetes as previously described for *B. burgdorferi* (25). Briefly, spirochetes grown in BSK II media to densities of ~10^7^/mL were pelleted, washed twice in PBS, and then sonicated in PBS. The protein concentration of the lysate was determined using the Bio-Rad Protein Concentration kit. One-hundred-microgram aliquots of lysate protein were applied to each 12% SDS-PAGE gel, and reactivity of sera with the separated proteins was analyzed by immunoblot as previously described for *B. burgdorferi* (25). To assess reactivity to a recombinant *B. miyamotoi* Vlp18 protein [generated as previously described (30)], 3 µg of purified protein was applied to a 12% SDS-PAGE gel for immunoblot analysis.

### Luminex assays

C6 peptides derived from the vlsE proteins of *Borrelia burgdorferi* (CMKKDDQIAAAIVLRGMAKDGQFVLD) and *Borrelia garinii* (CMKKDDQIAAAMVLRGMAKDGQFAL) were synthesized (GenScript) and chemically coupled to BSA. DNA sequences encoding the glpQ and fhbA antigens of *B. miyamotoi* (after removal of the signal peptide) were synthesized (Genewiz) and cloned into the pGEX-6p plasmid to direct the synthesis of a GST-antigen fusion protein. The GST moiety was cleaved by digestion with the PreScission protease, and the recombinant antigen was purified by a combination of glutathione affinity and ion-exchange chromatography. Vsp1, vlp5, vlp15/16, and vlp18 were prepared as previously described (30). Antigens were coupled to fluorescent beads at a concentration of 5–50 µg per million beads as recommended by the manufacturer (Luminex). Serum samples were diluted 1:400 in PBS, and antibodies binding to the antigen-coated beads were detected using a standard protocol recommended by the manufacturer (31). Each assay reaction contained 2,000 beads.

### Collection of cerebrospinal fluid

Mice were euthanized by CO_2_ asphyxiation prior to placement in a prone position on a stereotaxis frame with head fixed and angled downward at ~135°. Using a scalpel, a midline incision was made at the base of the skull, separating the subcutaneous (s.c.) tissues and neck muscles to expose the transparent dura mater covering the cisterna magna. Using a stereomicroscope, a pre-pulled sharp glass capillary was inserted through the dura to collect CSF via capillary action.

### Histopathology

For the preservation of mouse tissue in the absence of blood contamination, whole mouse perfusions were performed as previously described (32) except that 10% formalin instead of paraformaldehyde was used. Mice were euthanized as described above (see the *Mice* section) prior to perfusion and death confirmed prior to dissection according to American Veterinary Medical Association Guidelines for the euthanasia of animals, 2020 edition and Yale Institutional Animal Care and Use guidelines. Harvested tissues (brain, heart, tongue, esophagus, stomach, large and small intestines, liver, spleen, kidney, reproductive tract, pancreas, cervical and mesenteric lymph nodes, and salivary glands) were submitted to the Yale Comparative Medicine Pathology Research Core for standard FFPE processing and sectioning at 5 µm. Sections were stained with hematoxylin and eosin or Warthin–Starry according to established protocols (33). Light microscopic images were taken using a Zeiss Axioskop2 and with an Axiocam MrC camera.

## Results

### Identification and genetic analysis of *B. miyamotoi* clinical isolate CT14D4

Blood from a person with disseminated Lyme disease was inoculated i.p. into a B6 *Rag1*^−/−^ mouse. Within 2 weeks, a Giemsa stain of a blood smear revealed spirochetes (**Supplementary Figure 1**) and numerous spirochetes were visible by DF microscopy in blood samples collected over 7.5 months (**Supplementary Table 1**). PCRs of the *B. miyamotoi flaB* and *B. burgdorferi recA, ospA*, and 16SRNA genes were performed on infected mouse blood and only *B. miyamotoi flaB* DNA was detected (**Supplementary Table 1**).

**Figure 1:**
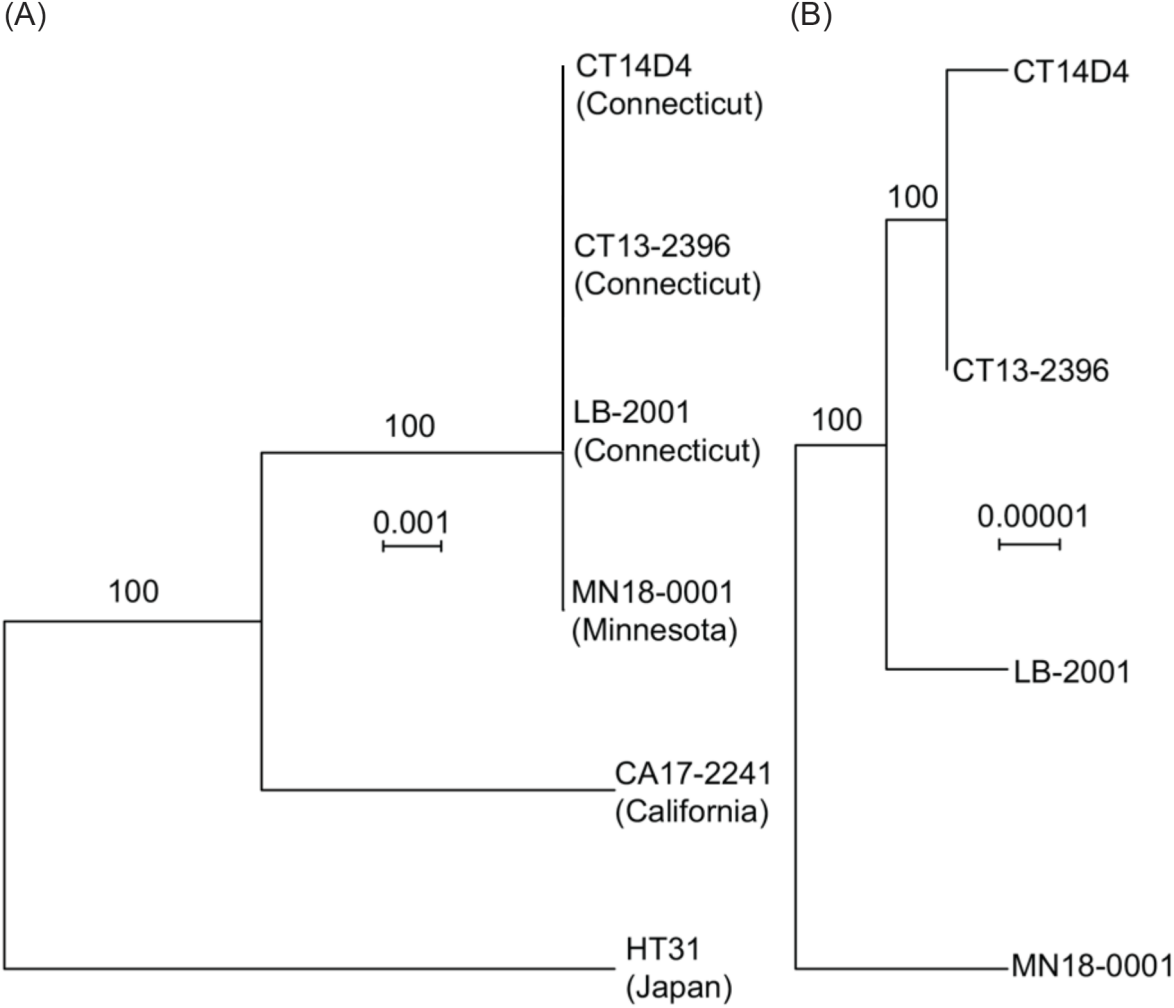
Distance phylograms of complete chromosome nucleotide sequences of CT14D4 and five other strains of *Borrelia miyamotoi*. The strain designations and geographic locations of their isolation are indicated. These (along with their GenBank accession numbers) were CT14D4 (CP010308), CT13-2396 (CP017126), LB-2001 (CP072479), MN18-0001 (CP150054), CA17-2241 (CP021872), and HT31 (AP024371). **(A)** Phylogram for the six chromosome sequences; **(B)** phylogram for chromosome sequences of three strains from Connecticut and one from Minnesota. Nucleotide sequences were aligned by MAFTT (https://mafft.cbrc.jp) with default settings. The observed distance phylograms were generated with SeaView version 4 (https://doua.prabi.fr/software/seaview) with 100 bootstrap iterations. The size bars in each panel are for the distance between any pair of sequences. The percent support for a node is indicated.

To further confirm that the spirochete isolate was *B. miyamotoi*, additional genetic analyses were performed. Of 903,819 ungapped aligned sites of the genome sequences, CT14D4 differed from the Connecticut strains LB-2001 (3) and CT13–2396 at 0.001% positions, from Minnesota strain MN-180001 at 0.005% positions, from California strain CA17–2241 at 1.1% positions, and from strain HT31 of Japan at 2.2% positions (**Figure 1**). The complete 16S ribosomal RNA gene and *flaB* gene sequences of CT14D4 were identical to those of strain LB-2001. The multilocus sequence type (MLST), based on partial sequences of eight housekeeping genes (https://pubmlst.org/organisms/borrelia-spp) (34), was ST634, the same as for LB-2001, but different from the sequence type for isolates of *B. miyamotoi* from Japan (ST633) and from Europe (ST635). These findings demonstrated that CT14D4 was almost identical to *B. miyamotoi* isolates from Connecticut, more divergent from an isolate from the north-central region of the United States but distinctly different from a representative of the *B. miyamotoi* of the far-western United States, as previously observed by MLST genotyping (35).

### Initial propagation of CT14D4 in B6 Rag1^−/−^ mice

Our initial efforts were directed toward propagation of CT14D4 and screening select WT and immunodeficient mouse strains for susceptibility to infection. Blood samples that contained spirochetes from the primary infected *Rag1*^−/−^ mouse were used to infect subsequent mice by i.p. inoculation, a route that we had previously used to infect mice with the soft tick relapsing fever (STRF) spirochete *B. hermsii* (36). In the first mouse passage, 100 µL of blood diluted 1:1 in Alsever’s solution (henceforth referred to as infected mouse blood) was collected from the primary B6 *Rag1*^−/−^ mouse at infection day 16 and inoculated into B6 *Rag1*^−/−^, C3H WT, and C3H *Myd88*^−/−^ mice (*N* = 1 each) (**Supplementary Table 1**). One month later, one B6 *Rag1*^−/−^ mouse was inoculated with infected mouse blood from the primary *Rag1*^−/−^ mouse collected on day 55 (resulting in another P1 passage) and a second with blood from the passage 1 (P1) *Rag1*^-/-^ mouse (resulting in a P2 passage). A third passage inoculated 1 B6 *Rag1*^-/-^, 2 B6.129 *FceRγ*^-/-^ and 1 B6 *Myd88*^-/-^ mice with 100 µl of pooled infected blood from the passage 0, 1 and 2 *Rag1^-/-^* mice. With the exception of B6 *Rag1*^−/−^ mice, which remained bacteremic until the termination of the experimental period (up to 30 weeks) or death, all other mice developed transient bacteremia that resolved within 10 days of inoculation (**Supplementary Table 1**). For a later passage, only B6 *Rag1*^−/−^ mice were inoculated. These mice expired more quickly than the primary infected *Rag1*^−/−^ mouse, with passage 4 infected mice succumbing to infection within 3 weeks. This may be due to increasing pathogen burdens in the donor-infected *Rag1*^−/−^ mice, the presence of multiple serotypes, and/or further host adaptation of CT14D4 as the population of spirochetes expanded in the absence of B cell selective pressure.

### Establishment of a tick–mouse infection cycle

**Figure 2:**
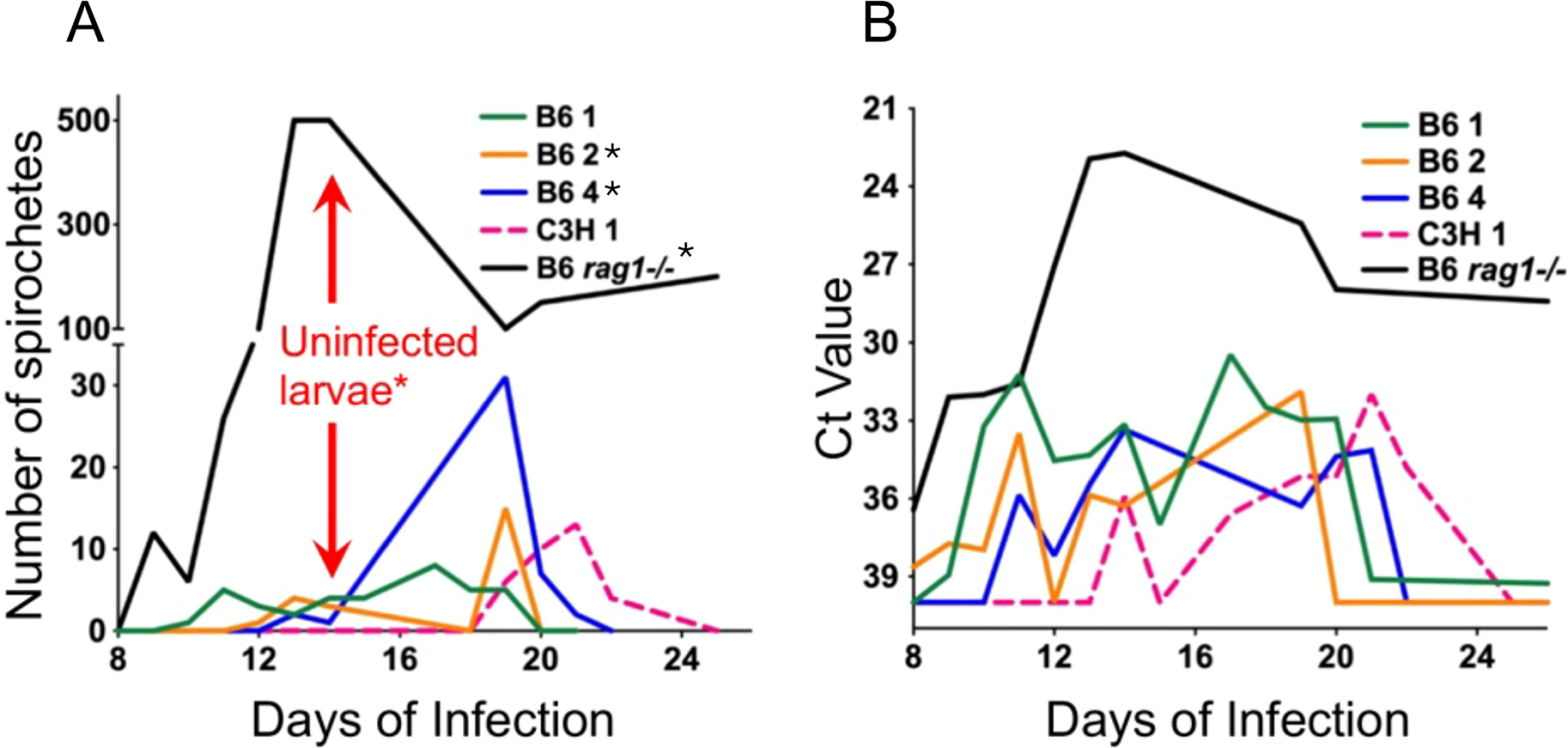
Assessment of bacterial burden by darkfield and quantitative PCR in mouse blood after nymphal transmission of CT14D4 (see **Table 1**). Only mice that acquired infection are shown. **(A)** Spirochetes enumerated in blood samples by darkfield; **(B)** CT values for quantitative PCR of the *B. miyamotoi flaB* gene in DNA isolated from blood. On day 14 of infection, uninfected larvae were placed on two of the B6 mice (note asterisks) and the B6 *Rag1^−/−^* mouse.

To assess whether CT14D4 could be passaged through a tick–mouse infection cycle, clean larvae were placed on the primary infected B6 *Rag1*^−/−^ mouse 18 days after inoculation with human blood. A representative sample of fed larvae analyzed by PCR 10 days after completion of the bloodmeal revealed that 3 of 12 larvae (25%) tested positive for *B. miyamotoi flaB* DNA (**Supplementary Figure 2**). The remaining larvae were allowed to molt to nymphs, at which time the prevalence of infected nymphs was estimated to be 10% by PCR. To assess whether the nymphs could transmit infection, 3 ticks were fed on each of five B6 WT, two B6 *Myd88*^−/−^ and five B6 *Rag1*^−/−^ mice (**Supplementary Table 2**). A transient low level of spirochetemia was detected on days 6 and 7 in a subset of infected B6 WT mice, with a higher spirochetemia detected on days 11–13. Only one of two *Myd88*^−/−^ mice became infected, with *B. miyamotoi* DNA detected by PCR of a blood sample collected on day 12 and visualized in a blood sample on day 13. By day 20 the WT and *Myd88*^−/−^ mice were no longer bacteremic whereas the B6 *Rag1*^−/−^ mice remained persistently infected. Immunosuppression of the WT or *Myd88*^−/−^ mice with cortisone did not result in resurgence of *B. miyamotoi* in blood as assessed by PCR and DF examination (data not shown). Analysis of the engorged nymphs retrieved after feeding to repletion revealed that mouse infection was observed if at least one of the retrieved ticks harbored *B. miyamotoi*, as assessed by DFA of ticks and PCR (**Supplementary Table 2**). In a second experiment, we compared infection of B6 with C3H/HeN mice using eight nymphs per mouse (**Table 1**; **Figure 2**). Results revealed that B6 mice appeared to be more susceptible to tick-transmitted infection than C3H mice, with the latter having a later onset of bacteremia. However, infection rates of all mice were lower than expected, which may be due to the extended duration (more than 4 months) between larval molting and nymphal feeding. All WT mice had resolved bacteremia by day 25 of infection and blood tested negative by DF and PCR at the end of the experimental period (day 49). To assess tick acquisition of *B. miyamotoi* infection, larval ticks were placed on two B6 WT mice and one *Rag1*^−/−^ mouse on day 14 of infection (**Figure 2**). After fed larvae molted to nymphs, PCR of a representative sample of ticks revealed that only 10% were positive (**Table 1**; **Figure 2**; **Supplementary Figure 3**), consistent with previous reports of low acquisition during larval feeding on laboratory mice and reduced survival of *B. miyamotoi* through molting to nymphs (37).

**Table 1:** Time course of spirochetemia after tick-transmitted infection.

Mouse strain/#	Days after tick attachment														Tick acquisition
	9	10	11	12	13	14	15	17	18	19	20	21	22	25	%PCR + (+/number ticks examined)
B6 1	–	1	5	3	2	2	4	8	5	5	–	–	nd	nd	na
*2	–	–	–	1	4	3	nd	–	15	–	–	–	nd	nd	10% (1/10)
3	–	–	–	–	–	–	–	–	–	–	–	nd	nd	nd	nd
*4	–	–	–	–	2	1	nd	nd	nd	31	7	2	–	nd	0% (0/5)
5	–	–	–	–	–	–	–	–	–	–	–	nd	nd	nd	na
C3H *1	–	–	–	–	–	–	–	–	–	6	10	13	4	–	na
2	–	–	–	–	–	–	–	–	–	–	–	–	nd	nd	na
3	–	–	–	–	–	–	–	–	–	–	–	–	nd	nd	na
4	–	–	–	–	–	–	–	–	–	–	–	–	nd	nd	na
5	–	–	–	–	–	–	–	–	–	–	–	–	nd	nd	na
*B6 Rag1^-/-^	12	6	26	100	500	500	nd	nd	nd	100	150	nd	nd	200	10% (1/10)

Spirochetes were enumerated by immunofluorescent staining of smears of plasma from blood samples obtained at the indicated time points. (-) indicates that no spirochetes were found. nd, not done; na, samples not available.

*Larvae were placed on the indicated mice at day 14 of infection.

PCR of the *B. miyamotoi flaB* gene was performed after larvae molted to nymphs. Results are expressed as the percent of nymphs testing positive and as the number of ticks testing positive over the number examined.

### Analysis of humoral responses during infection with CT14D4

**Figure 3:**
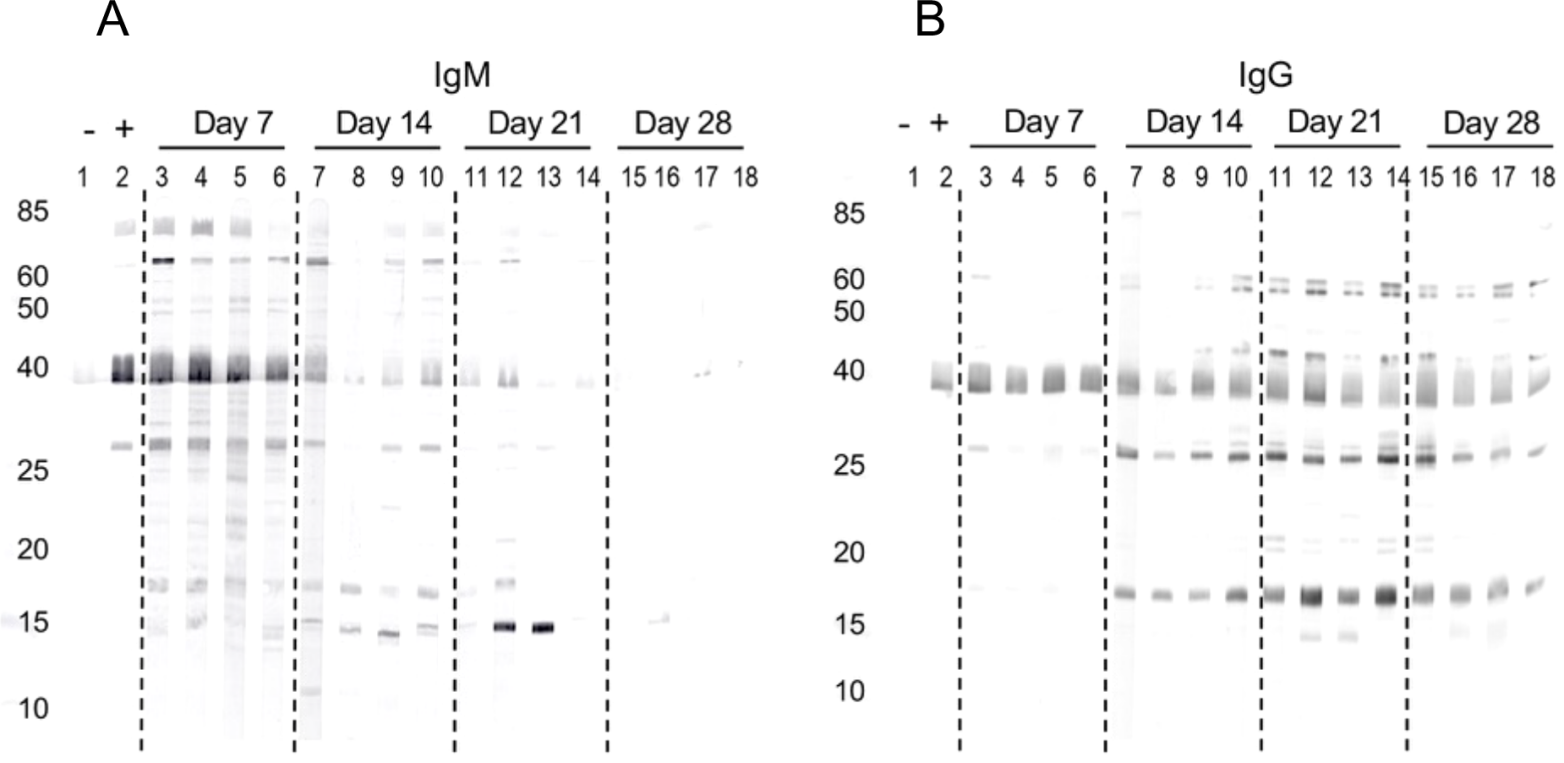
Time course of antibody responses in B6 WT mice infected with Tp2 spirochetes (see **Table 2**) assessed on CT14D4 lysate immunoblots. **(A)** IgM and **(B)** IgG. Lane 1, normal mouse sera; Lane 2, positive control CT14D4-infected mouse sera. Serial sera were collected from individual mice at the indicated time points. Mouse 1: lanes 3, 7, 11, and 15; Mouse 2: lanes 4, 8, 12, and 16; Mouse 3: lanes 5, 9, 13, and 17; Mouse 4: lanes 6, 10, 14, and 18. Because of space constraints on the blots, sera from four of five mice are shown. Vertical dotted lines are provided to separate samples drawn at the indicated time points.

**Figure 4:**
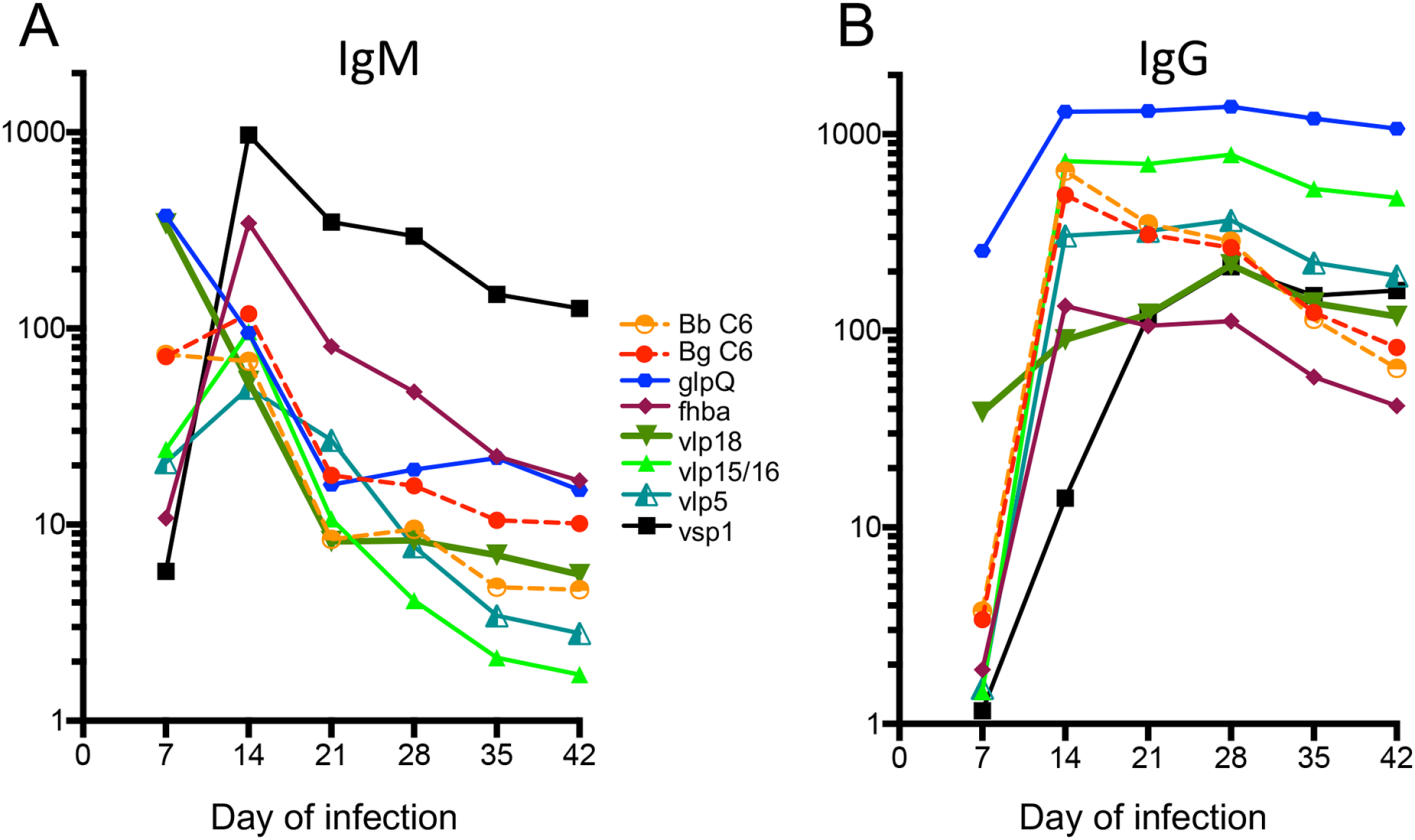
Responses to select *Borrelia* proteins over time in CT14D4-infected WT mice. Pooled sera from 5 B6 mice inoculated i.p. with infected mouse plasma were assessed at each time point. Luminex assays were used to detect **(A)** IgM and **(B)** IgG antibodies to the indicated recombinant antigens: Bb C6, the C6 peptide of the VlsE antigen from *B. burgdorferi*; Bg C6, the C6 peptide from *B. garinii* VlsE antigen; the remaining proteins are antigens from *B. miyamotoi*. Samples were tested at a 1:400 dilution; the average of two technical replicates is shown.

B cells and antibodies are known to be essential for controlling pathogen burdens during STRF infections (36, 38–40), with relapses of bacteremia reflecting emergence of spirochetes expressing a new Vmp/Vlp. To evaluate B-cell responses to *B. miyamotoi* antigens, we examined the evolution of IgM, IgG, and IgG subclass responses in infected B6 WT mice using both immunoblot of CT14D4 lysates and Luminex assay of specific proteins. Two sources of CT14D4 were used, one originally cultured from the blood of a WT B6 mouse infected by tick bite and the other from cryopreserved infected mouse plasma collected from the primary B6 *Rag1*^−/−^ mouse on day 16 (**Table 2**). As these samples were frozen and the number of viable spirochetes was low in the day 16 plasma sample, they were first inoculated into B6 *Rag1*^−/−^ mice to obtain fresh infected mouse plasma for inoculation into WT mice. Spirochetes were enumerated in blood samples from WT mice collected through 17 days of infection and were first detected by DF on day 3. On day 7, both IgM and IgG to *B. miyamotoi* proteins were detected on immunoblots, with predominant reactivity to protein(s) with a molecular size of ~40 kDa (**Figure 3**; **Supplementary Figure 4**). Luminex assays with recombinant proteins detected IgM to vlp18 on day 7 and vlp15/16 and vlp5 on day 14, all with molecular weights of ~40 kDa (**Figure 4A**). A vlp18 immunoblot (**Supplementary Figure 5**) confirmed reactivity to the vlp18 antigen. The IgM response to vlp18 waned by day 14 and was absent by day 21 (**Figures 3A**, **4A**), consistent with reduced IgM reactivity to a protein of that molecular weight on lysate immunoblot (**Figure 3A**; **Supplementary Figure 5**). These results are consistent with antigenic variation of Vmp/Vlp of *B. miyamotoi* (30, 41, 42) and demonstrate that vlp18 was a dominant antigen expression by cultured CT14D4. This is also consistent with published findings of early antibody responses to vlp18 in people infected with *B. miyamotoi* (43). The IgG response to proteins with a molecular weight of 40 kDa, first detected on day 7, remained elevated through day 28 (**Figure 3B**; **Supplementary Figure 4**). IgG reactivity to 40-kDa proteins was dominant on day 7, and by day 14, the antibody responses had expanded to multiple other proteins (**Figure 3B**). By Luminex assay, we found reactivity to fhba, glpQ, vsp1, and vlp5 as well as to the C6 peptide from *B. burgdorferi*, a cross-reactivity noted by others (**Figure 4B**) (44). Antibody responses were similar regardless of source of infecting spirochetes (**Figure 3**; **Supplementary Figure 4**). The reactivity of *B. miyamotoi*-specific IgG subclasses induced by infection revealed IgG2b and IgG2c responses with greater intensity on immunoblot than those of IgG1 and IgG3 after day 7 (**Supplementary Figure 6**).

**Table 2:** Kinetics of spirochetemia in mice infected with CT14D4 from different modes of propagation.

Mouse strain/number	Spirochete Source	DF enumeration of spirochetes						
		Day 3	Day 5	Day 7	Day 9	Day 11	Day 13	Day 17
B6 WT 1	Tp2	5/f^1^	–	1/ws^2^	3/ws	–	–	–
2	Tp2	2/f	–	14/ws	8/ws	1/ws	–	–
3	Tp2	5/f	–	3/ws	9/ws	–	–	–
4	Tp2	10/f	–	18/ws	–	–	–	–
5	Tp2	2/f	–	1/ws	–	5/ws	–	–
B6 *Rag1^-/-^* 1	Tp2	2/f	60/f	30/f	50/f	50/f	nd	nd

B6 WT 1	Plasma D16	1/3f	–	3/ws	8/ws	3/ws	–	–
2	Plasma D16	1/3f	–	1/ws	–	10/ws	–	–
3	Plasma D16	1/f	–	1/ws	49/ws	1/ws	–	–
4	Plasma D16	3/f	–	2/ws	9/ws	–	–	–
5	Plasma D16	1/3f	–	2/ws	22/ws	–	–	–
B6 *Rag1^-/-^* 2	Plasma D16	3/f	30/f	10/f	15/f	15/f	50/f	nd

Tp2 spirochetes were originally cultured from a tick-transmitted infection of a B6 WT mouse. Plasma D16 spirochetes were from a cryopreserved aliquot of infected mouse plasma collected at day 16 from the primary infected *Rag1^-/-^* mouse. Both were expanded in separate B6 *Rag1^-/-^* mice to obtain fresh plasma for infection.

^1^ f, visual field; ^2^ws, whole sample (30 visual fields); nd, not done

### Splenectomized mice have delayed clearance of CT14D4 infection

**Figure 5:**
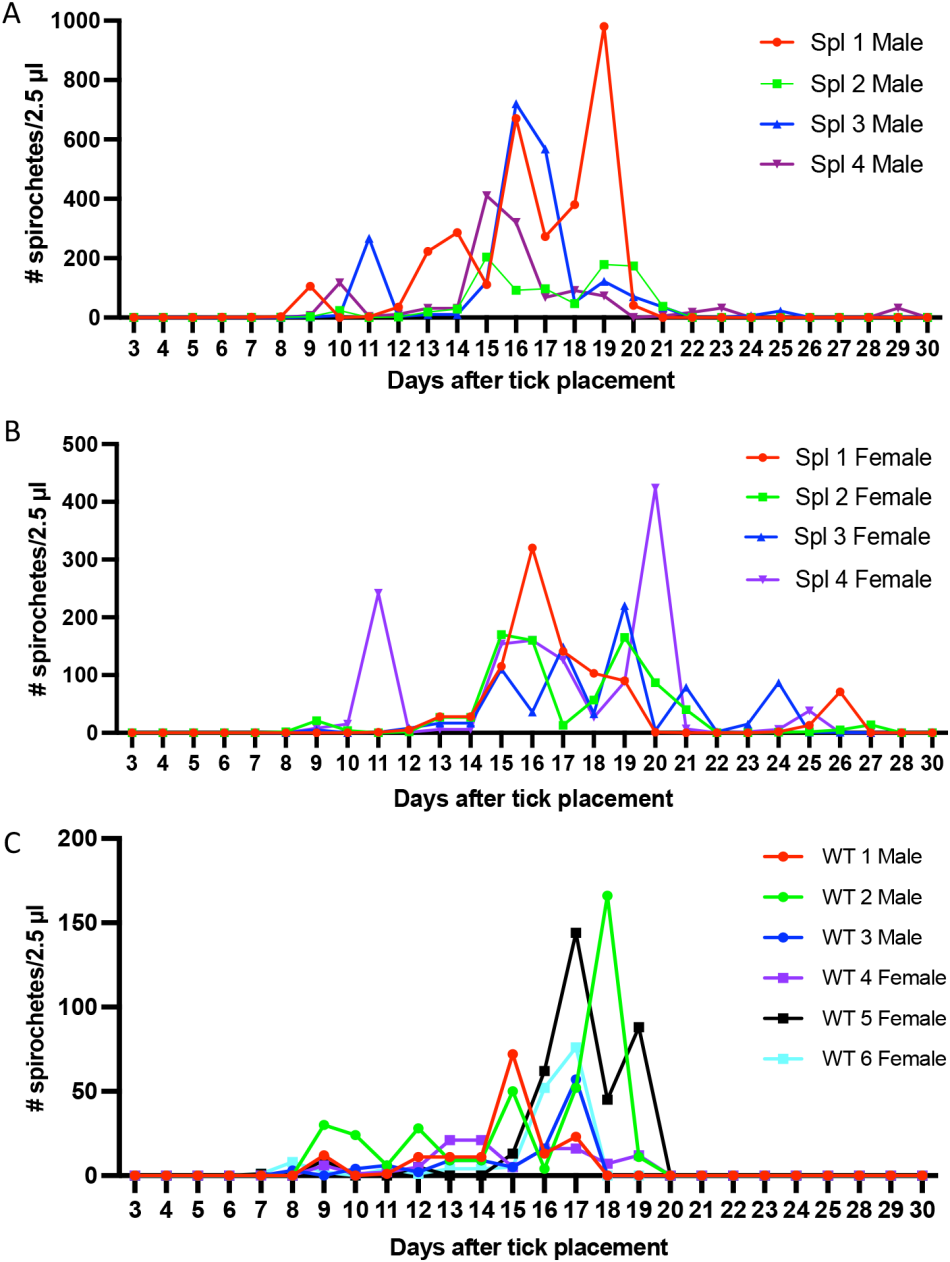
Kinetics of bacteremia in CT14D4 infection of splenectomized and WT B6 mice. Spirochetes were enumerated after DFA staining of plasma samples. **(A)** Male splenectomized mice; **(B)** female splenectomized mice; **(C)** male and female WT mice. Note differences in magnitude of episodes of bacteremia in the different groups.

Our initial screening experiments revealed that FcR and the TLR adaptor molecule MyD88 were dispensable for controlling spirochete burdens and clearing infection (**Supplementary Tables 1**, **2**). Since *B. miyamotoi* manifests as relapsing spirochetemia, we next sought to determine the contribution of the spleen to controlling pathogen burdens and clearance of spirochetes. B6 WT (three male and three female) and splenectomized mice (four male and four female) were infested with CT14D4-infected nymphal ticks, and spirochete burdens in the blood were determined by immunofluorescent staining and DF microscopy. No differences were observed in bacteremia during the first 8 days, but starting on day 9, the average number of spirochetes per stained blood smear was higher in the splenectomized mice (**Figure 5**). The spirochete burdens remained elevated past day 19, which was the last day spirochetes were detected in the blood of WT mice (**Figure 5C**). Splenectomized mice cleared spirochetes from the blood by day 30 (**Figures 5A, B**). There were no statistical differences between the spirochete burdens in male (**Figure 5A**) versus female (**Figure 5B**) mice although there was a trend towards more spirochetes in the male splenectomized mice.

**Figure 6:**
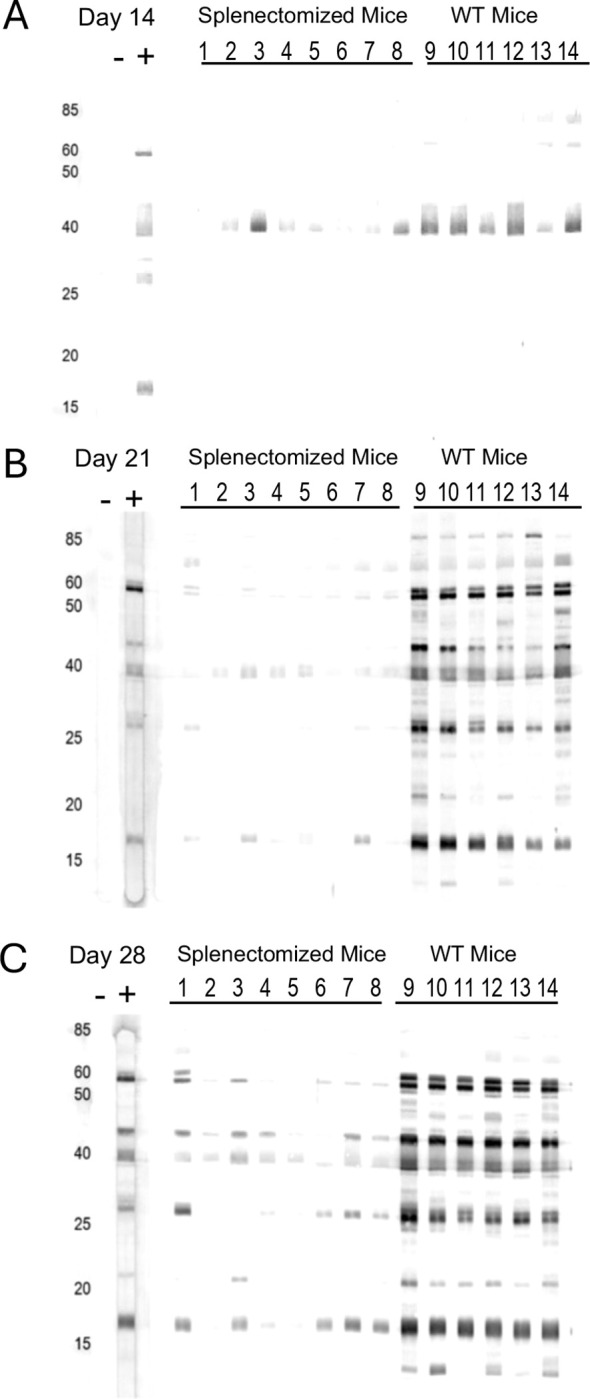
Time course of IgG responses in splenectomized and WT B6 mice infected by tick transmission assessed on CT14D4 lysate immunoblots. **(A)** Infection on day 14; **(B)** infection on day 21; **(C)** infection on day 28. Negative control, normal mouse sera; positive control, B6 WT infected mouse sera; lanes 1–4, splenectomized male mouse sera; lanes 5–8, splenectomized female mouse sera; lanes 9–11, WT male mouse sera; lanes 12–14, WT female mouse sera.

Immunoblot analysis of the IgG response to *B. miyamotoi* over time demonstrated reduced IgG responses through day 28 after tick infestation in splenectomized mice, with reactivity to fewer antigens in comparison to WT mice (**Figure 6**). Between days 14 and 28, the intensity of the IgG responses modestly increased on immunoblot, but the number of antigens to which antibody responses were detected remained limited in comparison to WT mice (**Figure 6**). The increase in antibody responses correlated with the eventual clearance of the spirochetes in the splenectomized mice.

### Immunocompetent mice infected with CT14D4 may resist reinfection

**Figure 7:**
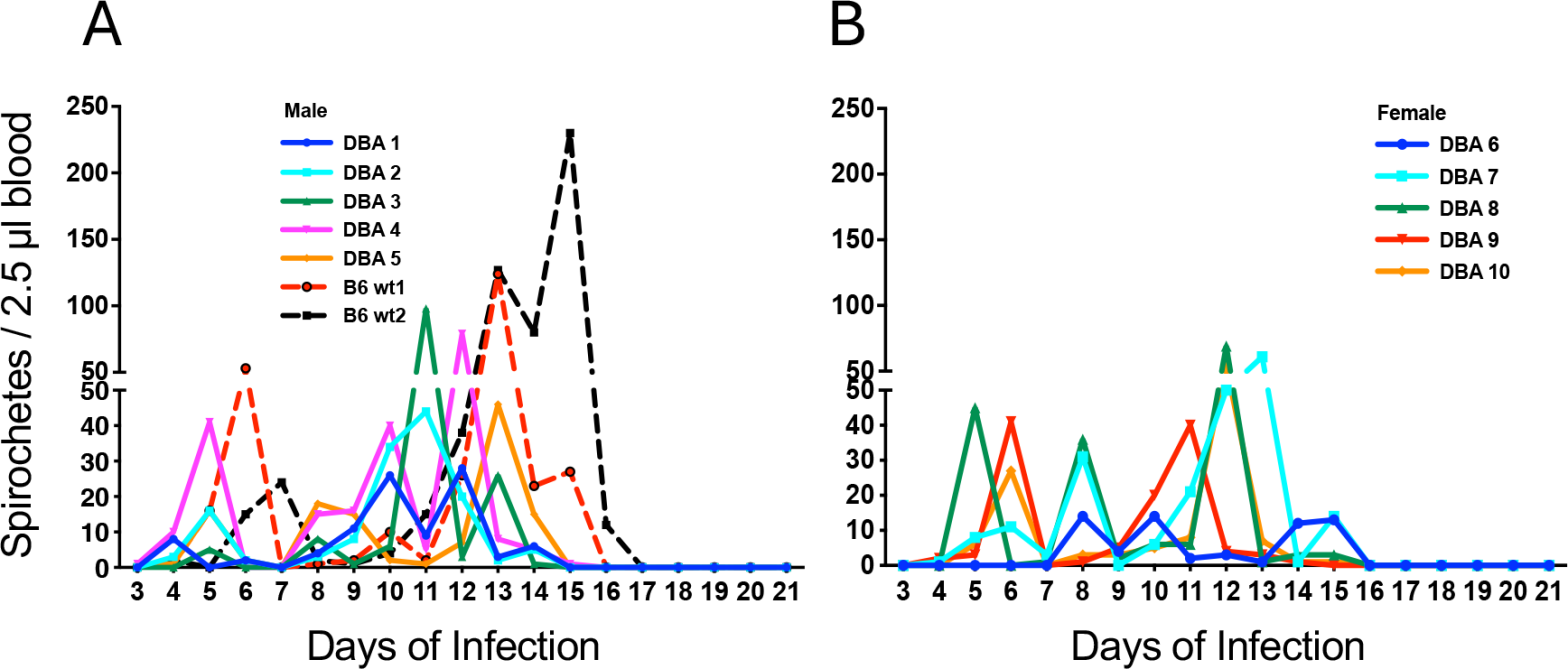
Spirochete burdens in the blood of CT14D4-infected DBAJ1 (DBA) mice vs. B6 WT. Mice were infected by s.c. inoculation with ~10^4^ spirochetes in freshly isolated infected mouse plasma. Spirochetes were enumerated by DFA, and the total number of spirochetes detected in plasma from 2.5 μL of blood are shown for individual mice. **(A)** Male mice, **(B)** female mice.

To determine whether mice infected with CT14D4 become resistant to reinfection, we challenged mice with CT14D4 after infection had resolved. A pilot experiment with infected B6 mice and C3H mice (*N* = 2 each strain) that had resolved bacteremia by day 20 rechallenged mice with infected mouse plasma on day 49. No spirochetes were observed in the blood over the next 30 days or at the time of sacrifice on day 82 (data not shown). PCR of tissues, however, revealed that the brain tissue from one of the B6 mice tested positive for *B. miyamotoi flaB* DNA (**Supplementary Figure 7**). Whether this was residual from the primary infection or a consequence of the challenge is unclear. The absence of demonstrable bacteremia after challenge suggests the former. We also conducted in parallel an experiment in DBA1J mice and B6 mice, as a previous study suggested that DBA1J mice control spirochetemia better than B6 mice after infection with the STRF spirochete *B. hermsii* (40). Mice were inoculated subcutaneously with B6 *Rag1*^−/−^ mouse plasma containing 10^4^ spirochetes. DBA1J mice exhibited earlier onset and more episodes of spirochetemia than B6 mice (3 vs. 2), but the peak bacteremia trended higher in the B6 mice, and they took a day or two longer than DBA1J mice to clear bacteremia (**Figure 7A**). Similar to findings in B6 splenectomized mice, spirochete burdens in male mice trended higher than in female mice (**Figures 7A, B**). Twelve days after bacteremic episodes had resolved, we challenged these mice by s.c. inoculation of infected mouse plasma containing 10^4^ spirochetes. Spirochetemia was assessed daily for 39 days after challenge and no spirochetes were detected in the blood of any of the mice at any of the time points (data not shown). Tissue PCR was not performed on these mice. These findings suggest that CT14D4 infection of WT mice can result in immunity that prevents reinfection of the same host. It is possible, however, that infection of the brain may occur during the primary infection and persist after bacteremia is cleared, as has been shown with STRF spirochetes (45–47).

### Tissue pathology reveals *B. miyamotoi* disease

**Figure 8:**
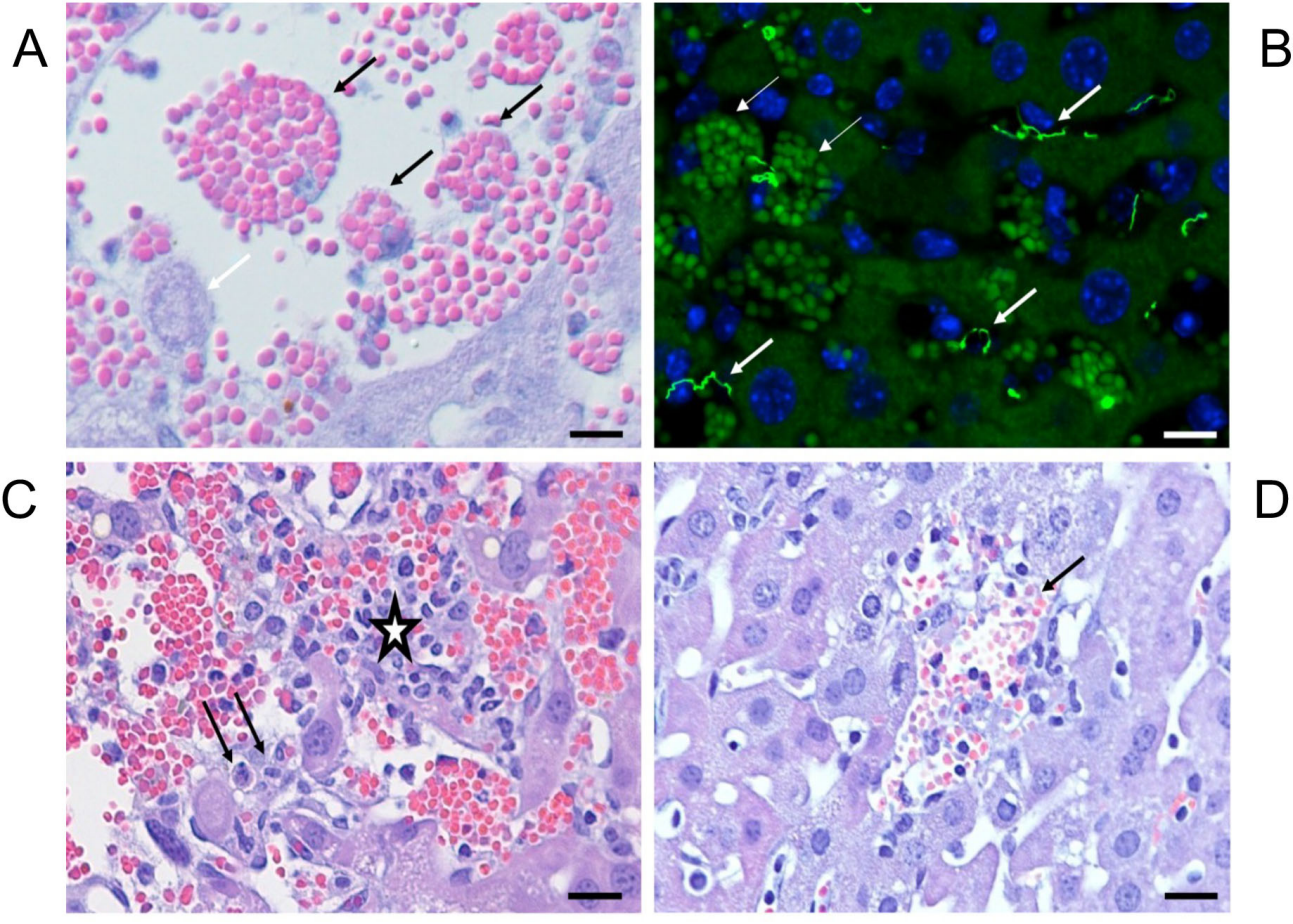
Hepatic pathology in B6 *Rag1^−/−^* **(A–C)** and WT **(D)** mice. **(A)** Sinusoids are characterized by marked distension, erythrophagocytosis (black arrows), and loss of sinusoidal endothelium and hepatocyte fragmentation (white arrow). **(B)** Spirochetes can be seen within sinusoids (thick arrows) and associated with phagocytosed erythrocytes (fine arrows). This is accompanied by significant hepatic pathology **(C)** characterized by hepatocellular death and disruption of hepatic plates (black arrows) and localized mononuclear hepatitis (☆). **(D)** Sinusoidal pathology (arrow) in WT mice is qualitatively similar, but far less severe. **(A, C, D)** Hematoxylin and eosin staining; **(B)** *B. miyamotoi* immunofluorescence staining after perfusion fixation of mice and antigen retrieval performed on FFPE liver section. Bar = 20 μm.

**Figure 9:**
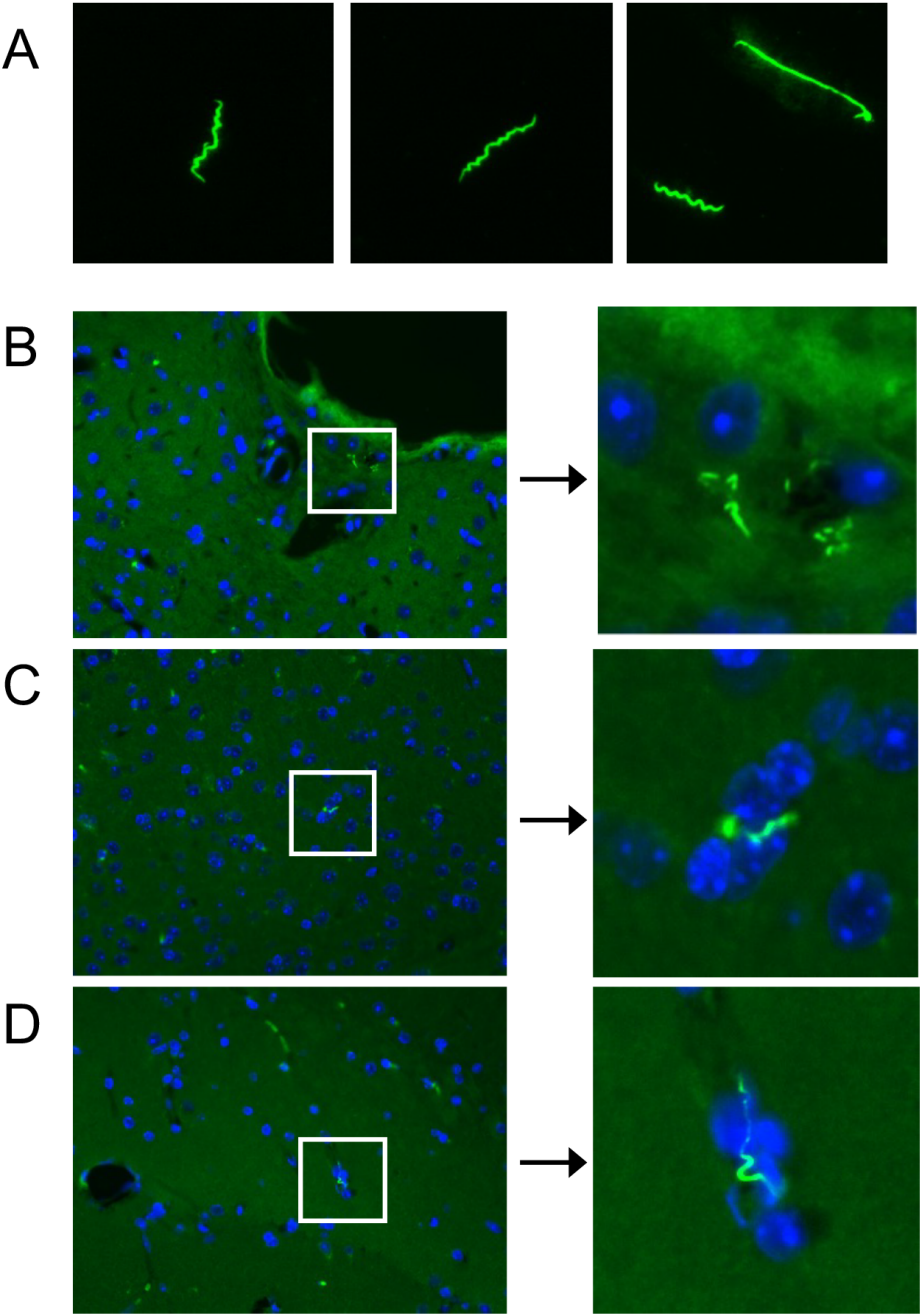
Immunofluorescent staining of CT14D4 in CSF **(A)** and in perfused FFPE B6 *Rag1^−/−^* mouse brain sections after antigen retrieval **(B–D)**. Images in **(A)** were originally obtained at 40× magnification and enlarged twofold after cropping. Images in **(B)** were obtained from a brain tissue section of a B6 *Rag1^−/−^* mouse at 40×. Images in **(C)** and **(D)** were obtained from a second *Rag1^−/−^* mouse in a different experiment. Images in panels **(B)** to **(D)** were obtained at 40× magnification with areas in white boxes enlarged to better visualize spirochetes.

We examined whether CT14D4 infection is associated with disease by performing histopathology on tissues of infected mice after perfusion fixation. Infected *Rag1*^−/−^ mice developed a multisystemic inflammatory pathology most striking in the liver and independent of infection route (inoculation vs. tick-transmission) (**Figures 8A–C**). Florid sinusoidal dilation and erythrophagocytosis were accompanied by mononuclear hepatitis, vasculitis, and thrombosis after infection introduced using CT14D4-infected mouse plasma. Hepatic and splenic extramedullary hematopoiesis was marked with some erythrophagocytosis evident in the latter organ. Histiocytic inflammation and thromboses were evident in pulmonary parenchyma and, to a lesser extent, in the heart (**Supplementary Figure 8**). Bacteremic WT mice develop qualitatively similar but far less severe hepatic lesions (**Figure 8D**) and had traces of inflammatory changes in other sites (**Supplementary Figure 9**). Splenectomized mice did not have more severe hepatic lesions than WT mice (data not shown). Spirochetes were visualized in the CSF of *Rag1*^−/−^ mice sampled immediately after euthanasia and also in the brain parenchyma after perfusion fixation (**Figure 9**). Other organs that exhibited mild pathology included the heart and pancreas but not kidneys or urinary bladders (**Supplementary Figure 8**, **9**).

## Discussion

We have established a mouse model of *B. miyamotoi* infection using the clinical *B. miyamotoi* isolate CT14D4. Genetic sequencing demonstrates that CT14D4 is virtually identical in its chromosome to two strains isolated from Connecticut ticks (3). Unlike these other two strains, our studies with CT14D4 show that this pathogen can infect multiple laboratory strains of WT mice, be acquired and transmitted by feeding ticks, and cause pathology in several organ systems, making it a suitable strain to study *B. miyamotoi* pathogenesis and disease in laboratory mice.

Previous studies using *B. miyamotoi* strains LB2001 and CT13-2396 (both isolates from ticks in CT) provided initial insights into host defenses required to control bacteremia (48). Infection of SCID mice with these strains resulted in high levels of spirochetemia that persisted for weeks to months, depending on the experimental time period examined, implicating T- and B-cell immunity in host defense. Larvae feeding on the infected mice could acquire infection, although the prevalence of infected ticks declined with each molt, from larvae to nymphs and nymphs to adults. The LB2001 strain in our laboratory had lost the ability to infect ticks, possibly due to serial passage in SCID mice, whereas CT14D4 can be both acquired and transmitted by ticks. Despite feeding on immunodeficient *Rag1*^−/−^ mice with high pathogen burdens, larval infection rates with CT14D4 were low. Vertical transmission from an infected adult tick to the egg clutch has been shown to be more efficient than horizontal transmission of *B. miyamotoi* (37), with pathogen burden in the adult tick correlating with percentages of larvae infected (49). Since completion of the studies reported herein, we have established that CT14D4-infected nymphs retain the infection after molting to adults. Infection rates of larvae hatched from egg clutches produced by adult females after mating and feeding on rabbits can exceed 70% (**Supplementary Figure 10**). Nymphs retain this rate of infection and are highly efficient at infecting new mice^1^.

LB2001 and CT13–2396 can infect WT mouse strains, including outbred CD1 mice and C3H/HeJ mice, which lack signaling through TLR4 (50). Infections were self-limiting, typically resolving within 2 weeks after introduction of spirochetes. Of these mouse strains, C3H/HeJ appeared the most susceptible, with both PCR and DF analysis of blood positive at multiple time points after infection. However, neither LB2001 nor CT13–2396 could be detected in mouse organs (heart, joint, and urinary bladder) by PCR, and larvae feeding on infected mice tested negative for *B. miyamotoi* DNA. Nymphs molted from these larvae also tested negative and did not transmit infection to naïve mice. These results contrast with our findings using CT14D4. Using multiple routes of infection, including i.p. or s.c. inoculation of blood or plasma from an infected *Rag1*^−/−^ mouse and tick transmission, we found that several WT mouse strains (C57BL/6J, C3H/HeN, and DBA1J) could be infected with this clinical isolate. WT mouse strains experienced multiple (typically ≤3) episodes of bacteremia that were visualized on blood smears, with clearance of infection within 4 weeks. As expected, the kinetics of bacteremia onset differed with route of infection, with mice taking longer to become bacteremic with tick-transmitted infection (within 8–9 days) than after i.p. or s.c. inoculation of spirochetes, consistent with reported tick transmission dynamics of *B. miyamotoi* infection (51). We found that CT14D4 DNA could be detected by PCR in the blood during episodes of bacteremia in B6 WT mice but not in the heart, spleen, liver, or urinary bladders. The differences observed between CT14D4 and its closely related isolates LB2001 and CT13–2396 may be due to differences in their chromosomal or plasmid sequences (5), original sources (mammalian isolate versus tick isolate), modes of propagation after isolation, and/or differences in the vector competency of the *I. scapularis* ticks used in our studies (52).

Infection of mouse strains with different immunodeficiencies revealed divergent mechanisms of immune control of *B. miyamotoi* compared to STRF spirochetes. In B6 *Rag1*^−/−^ mice infected with CT14D4, small numbers of spirochetes could be detected within the first 6 days of infection with a slight dip before a more sustained increase in bacteremia. The initial episode of bacteremia in CT14D4-infected WT mice was also smaller than subsequent relapses. This contrasts with the patterns seen with the STRF spirochete *Borrelia hermsii.* Infections of B6 *Rag2*^−/−^ and Balb/c *Rag* 2^−/−^ mice with *B. hermsii* reveal that the initial peak in bacteremia occurs within the first 5 days after inoculation and subsequently declines, only to rise again after day 10 (40). The decline in the initial peak implicated not only B cells but also innate immune mechanisms independent of antibody as factors contributing to host defense in early infection. Subsequent studies have confirmed a role for T-independent antibodies, especially those produced by B1b cells, as critical for clearance of *B. hermsii*, and also have demonstrated a requirement for MyD88-mediated TLR signaling (38, 39). *B. hermsii* infection of B6 *Myd88*^−/−^ mice results in enhanced pathogen burden and death within 16 days. Mice develop severe hepatosplenomegaly. IgM reactivity to proteins of lower molecular weight, typical of vsps, is lower the first week of infection in comparison to WT mice. Similar susceptibility to elevated pathogen burdens was observed in *B. hermsii*-infected *TLR2*^−/−^ mice. This contrasts with the finding that *TLR2*^−/−^ mice infected with a related STRF spirochete, *Borrelia turicatae*, exhibit similar pathogen burdens and disease as seen in WT mice, implying variability in requirements for host immunity other than B cells among different STRF spirochetes (47). Although examined in only a limited number of mice, we observed that CT14D4-infected *Myd88*^−/−^ mice cleared bacteremia, had comparable spirochete burdens to immunocompetent mice after needle inoculation or tick-transmitted infection, and did not develop hepatosplenomegaly. These results suggest that MyD88-mediated innate immune defenses, including signaling through TLR4 as seen with infection of C3H/HeJ mice (50), are dispensable for the clearance of *B. miyamotoi* and imply a greater reliance on B cell-mediated immunity.

As with other RF spirochetes, *B. miyamotoi* has been shown to undergo antigenic variation of its Vmps in mice (41). The CT14D4 used in our studies has not been cloned, although we consistently see that the predominant early antibody response is directed toward Vlps, including vlp18. IgM and weaker IgG responses to antigens in the 40-kDa range were detectable on day 7 of infection, with reactivity to multiple vsps and other antigens thereafter. Our results also show that all IgG isotypes are generated against CT14D4 antigens. The B-cell sources of these antibodies remain to be explored. Our experiments with splenectomized mice demonstrate a contribution of the spleen to the production of early antibodies against CT14D4. IgG to CT14D4 antigens are reduced during the first 4 weeks of infection in these mice, with clearance of spirochetes correlated with increasing intensity of IgG reactivity on immunoblots. While the spleen may contribute to the early control of CT14D4 infection, eventually extra-splenic antibody production can clear spirochetes from the blood. Our challenge experiments with B6, C3H, and DBA1J mice provide evidence that these previously infected mice are protected against reinfection, although the brain (as found in a single B6 mouse) could serve as a site of persistence despite antibodies. As noted earlier, B1b cells, which reside in the peritoneal cavity, are the main subset providing long-lasting protection against *B. hermsii* infection (39). Although B1b cells can produce both IgM and IgG3 antibodies, IgM alone was sufficient to confer immunity against *B. hermsii*. For the STRF spirochete *Borrelia duttoni*, IgM and IgG3 were found to be the most important isotypes that conferred protection against spirochetemia and death (53). Additional studies are required to determine whether the same holds true for *B. miyamotoi* infection.

Severe liver tissue pathology is one of the most striking consequences of high *B. hermsii* burdens in the absence of host B and T cells (40, 54). We observed that pathology in CT14D4-infected B6 *Rag1*^−/−^ mice had very similar findings of hepatitis, sinusoidal dilation, and marked erythrophagocytosis. Spirochetes were visualized in the tissues but were not associated with hepatosplenomegaly that is characteristic of *B. hermsii*-infected mice. Elevated hepatic transaminases have been found in people diagnosed with *B. miyamotoi* infection (55). We also found inflammatory infiltrates in multiple other organs in *Rag1*^−/−^ mice, including the lung, heart, pancreas, and mesenteric fat (**Supplementary Figure 8**). These changes were present in WT mice but to a lesser degree (**Supplementary Figure 9**). Although rare spirochetes could be visualized in CSF samples and in the brain of *Rag1*^−/−^ mice, they were not associated with pathology (**Figure 9**). We did not observe spirochetes in WT mouse brains after perfusion fixation. STRF spirochetes are known to cross the blood–brain barrier and can remain long-term as a silent infection even when spirochetes are no longer detected in the blood (45, 46, 56). Treatment with ceftriaxone can eliminate spirochetes from the brain (57). *B. miyamotoi* central nervous system infection presenting as meningoencephalitis has been reported in immunocompromised people, especially those who have been treated with B cell-depleted immunotherapies (18, 21, 24, 58–61). Future studies are warranted to examine the potential for CT14D4 to penetrate into the brain of WT mice or persist at that site after resolution of bacteremia.

The results of our studies are limited in part by our enumeration of spirochetes by DF microscopy or direct immunofluorescent staining rather than quantification by qPCR. We found that in comparison to microscopy, our qPCR assay was not sufficiently sensitive to detect CT14D4 in the small amounts of blood obtained from frequent tail vein bleeds. Microscopy enumeration permitted detection of qualitative changes in pathogen burden in WT mice and were critical for coordinating studies of pathology with periods of bacteremia.

In summary, we have established a mouse model of *B. miyamotoi* infection and disease. Our isolation of CT14D4 from the blood of a person with clinical signs of Lyme disease has demonstrated that *B. miyamotoi* co-infection with Lyme *Borrelia* may occur. The person from whom CT14D4 was isolated did not seroconvert to CT14D4 or to *B. burgdorferi*, presumably due to early intervention with doxycycline. The results of our studies show that this clinical isolate is a suitable strain to study *B. miyamotoi* pathogenesis in laboratory mice and reproduces some of the manifestations of BMD seen in human infection.

## Data Availability

The sequence of the chromosome of B. miyamotoi CT14D4 is available under GenBank accession number CP010308.

## Footnotes

^1^Belperron, et al. *Borrelia miyamotoi* negative impacts pregnancy in immunodeficient mice. Front Immunol. submitted.
