# Supplementary material for: Establishment of a laboratory mouse model to study Borrelia miyamotoi infection and disease
Source: Front Immunol. 2026 Jun 12;17:1851617. doi: 10.3389/fimmu.2026.1851617 (PMC13303035; doi:10.3389/fimmu.2026.1851617)
Supplement: Supplementary file 1 [file Presentation1.pptx]

## Slide 1
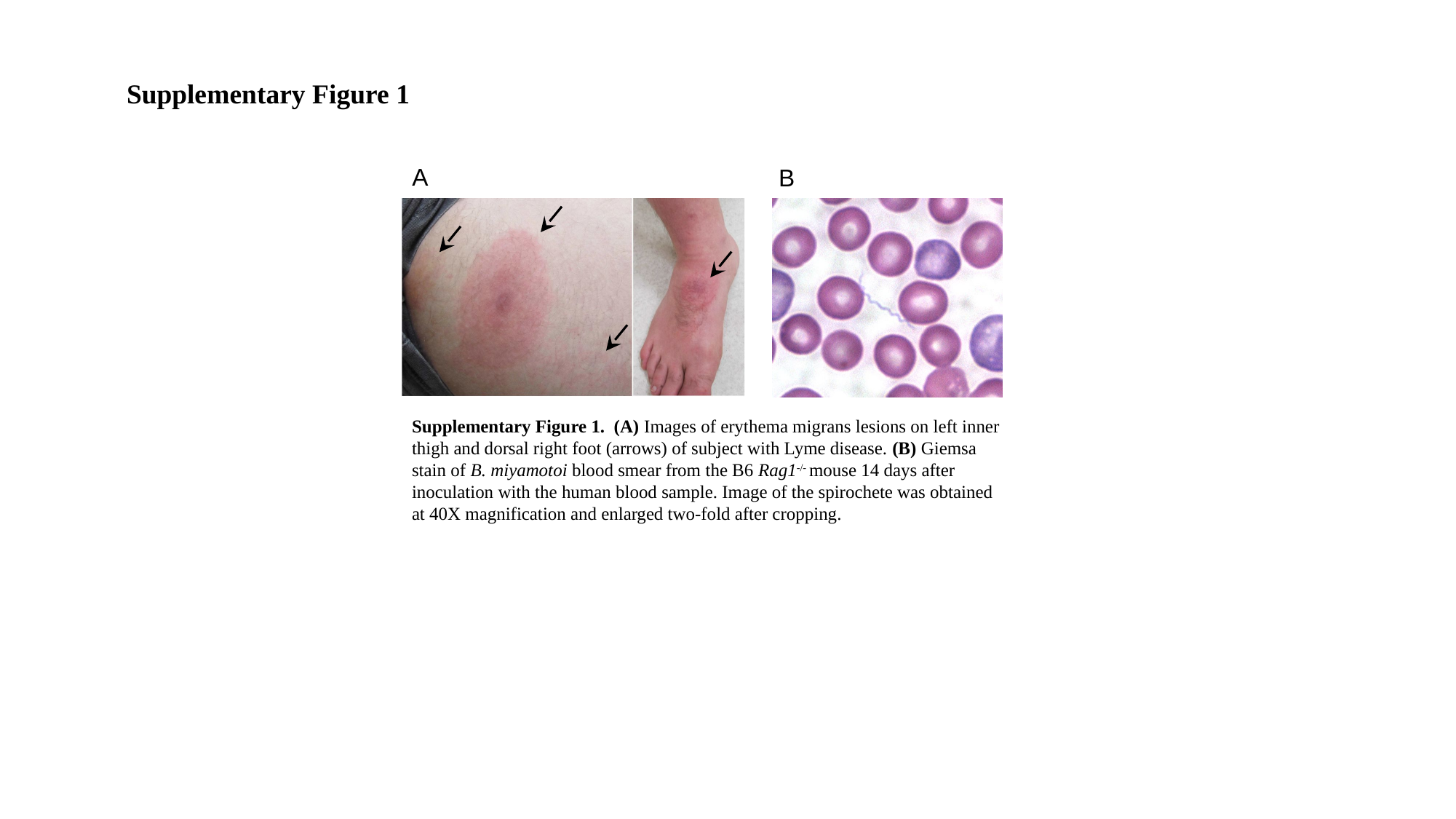

Supplementary Figure 1
A
B
Supplementary Figure 1. (A) Images of erythema migrans lesions on left inner thigh and dorsal right foot (arrows) of subject with Lyme disease. (B) Giemsa stain of B. miyamotoi blood smear from the B6 Rag1-/- mouse 14 days after inoculation with the human blood sample. Image of the spirochete was obtained at 40X magnification and enlarged two-fold after cropping.

## Slide 2
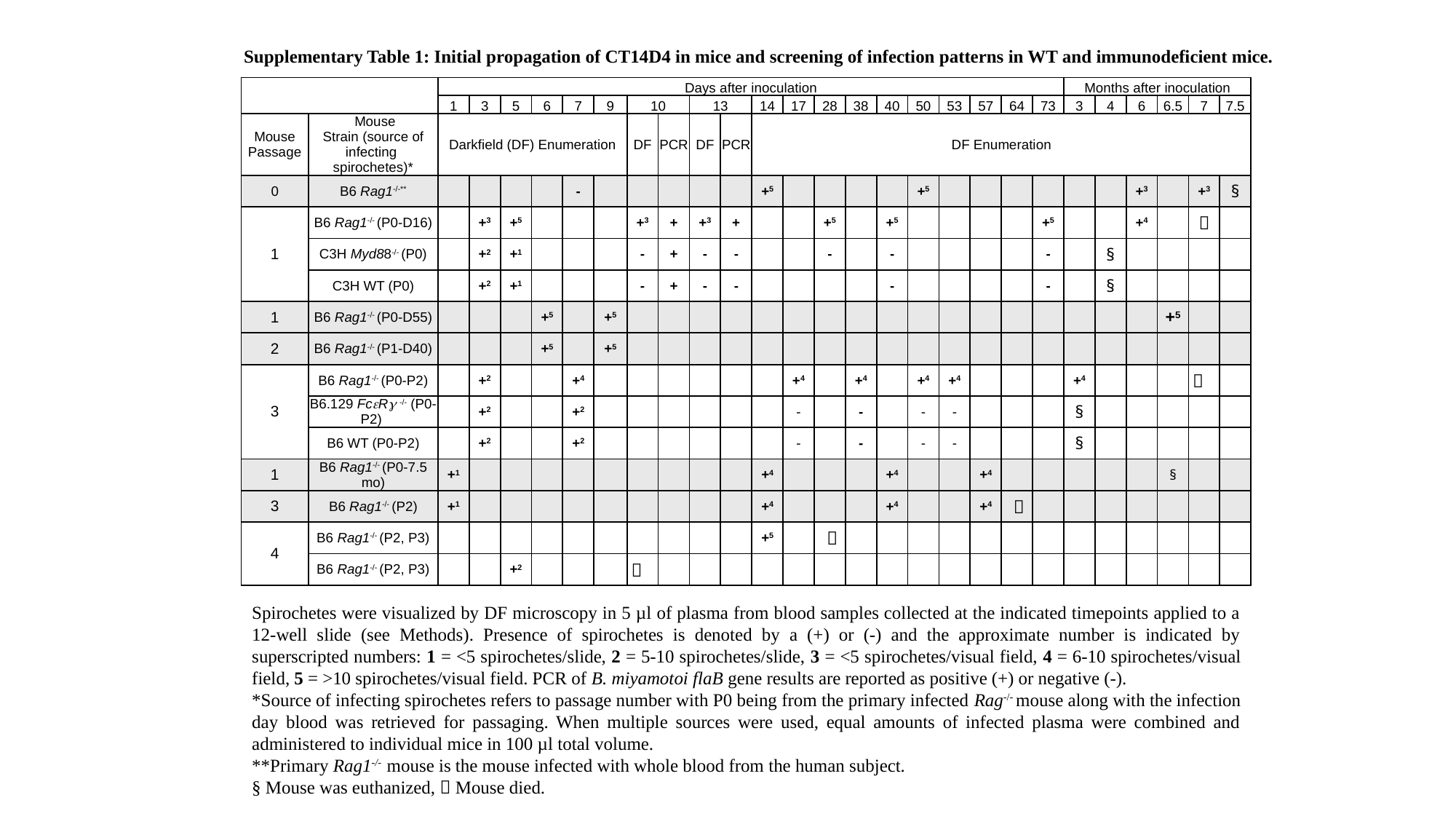

Supplementary Table 1: Initial propagation of CT14D4 in mice and screening of infection patterns in WT and immunodeficient mice.
| | | Days after inoculation | | | | | | | | | | | | | | | | | | | | Months after inoculation | | | | | |
| --- | --- | --- | --- | --- | --- | --- | --- | --- | --- | --- | --- | --- | --- | --- | --- | --- | --- | --- | --- | --- | --- | --- | --- | --- | --- | --- | --- |
| | | 1 | 3 | 5 | 6 | 7 | 9 | 10 | | 13 | | 14 | 17 | 28 | 38 | 40 | 50 | 53 | 57 | 64 | 73 | 3 | 4 | 6 | 6.5 | 7 | 7.5 |
| Mouse Passage | Mouse Strain (source of infecting spirochetes)\* | Darkfield (DF) Enumeration | | | | | | DF | PCR | DF | PCR | DF Enumeration | | | | | | | | | | | | | | | |
| 0 | B6 Rag1-/-\*\* | | | | | - | | | | | | +5 | | | | | +5 | | | | | | | +3 | | +3 | § |
| 1 | B6 Rag1-/- (P0-D16) | | +3 | +5 | | | | +3 | + | +3 | + | | | +5 | | +5 | | | | | +5 | | | +4 | |  | |
| | C3H Myd88-/- (P0) | | +2 | +1 | | | | - | + | - | - | | | - | | - | | | | | - | | § | | | | |
| | C3H WT (P0) | | +2 | +1 | | | | - | + | - | - | | | | | - | | | | | - | | § | | | | |
| 1 | B6 Rag1-/- (P0-D55) | | | | +5 | | +5 | | | | | | | | | | | | | | | | | | +5 | | |
| 2 | B6 Rag1-/- (P1-D40) | | | | +5 | | +5 | | | | | | | | | | | | | | | | | | | | |
| 3 | B6 Rag1-/- (P0-P2) | | +2 | | | +4 | | | | | | | +4 | | +4 | | +4 | +4 | | | | +4 | | | |  | |
| | B6.129 FceRg -/- (P0-P2) | | +2 | | | +2 | | | | | | | - | | - | | - | - | | | | § | | | | | |
| | B6 WT (P0-P2) | | +2 | | | +2 | | | | | | | - | | - | | - | - | | | | § | | | | | |
| 1 | B6 Rag1-/- (P0-7.5 mo) | +1 | | | | | | | | | | +4 | | | | +4 | | | +4 | | | | | | § | | |
| 3 | B6 Rag1-/- (P2) | +1 | | | | | | | | | | +4 | | | | +4 | | | +4 |  | | | | | | | |
| 4 | B6 Rag1-/- (P2, P3) | | | | | | | | | | | +5 | |  | | | | | | | | | | | | | |
| | B6 Rag1-/- (P2, P3) | | | +2 | | | |  | | | | | | | | | | | | | | | | | | | |
Spirochetes were visualized by DF microscopy in 5 µl of plasma from blood samples collected at the indicated timepoints applied to a 12-well slide (see Methods). Presence of spirochetes is denoted by a (+) or (-) and the approximate number is indicated by superscripted numbers: 1 = <5 spirochetes/slide, 2 = 5-10 spirochetes/slide, 3 = <5 spirochetes/visual field, 4 = 6-10 spirochetes/visual field, 5 = >10 spirochetes/visual field. PCR of B. miyamotoi flaB gene results are reported as positive (+) or negative (-).
*Source of infecting spirochetes refers to passage number with P0 being from the primary infected Rag-/- mouse along with the infection day blood was retrieved for passaging. When multiple sources were used, equal amounts of infected plasma were combined and administered to individual mice in 100 µl total volume.
**Primary Rag1-/- mouse is the mouse infected with whole blood from the human subject.
§ Mouse was euthanized,  Mouse died.

## Slide 3
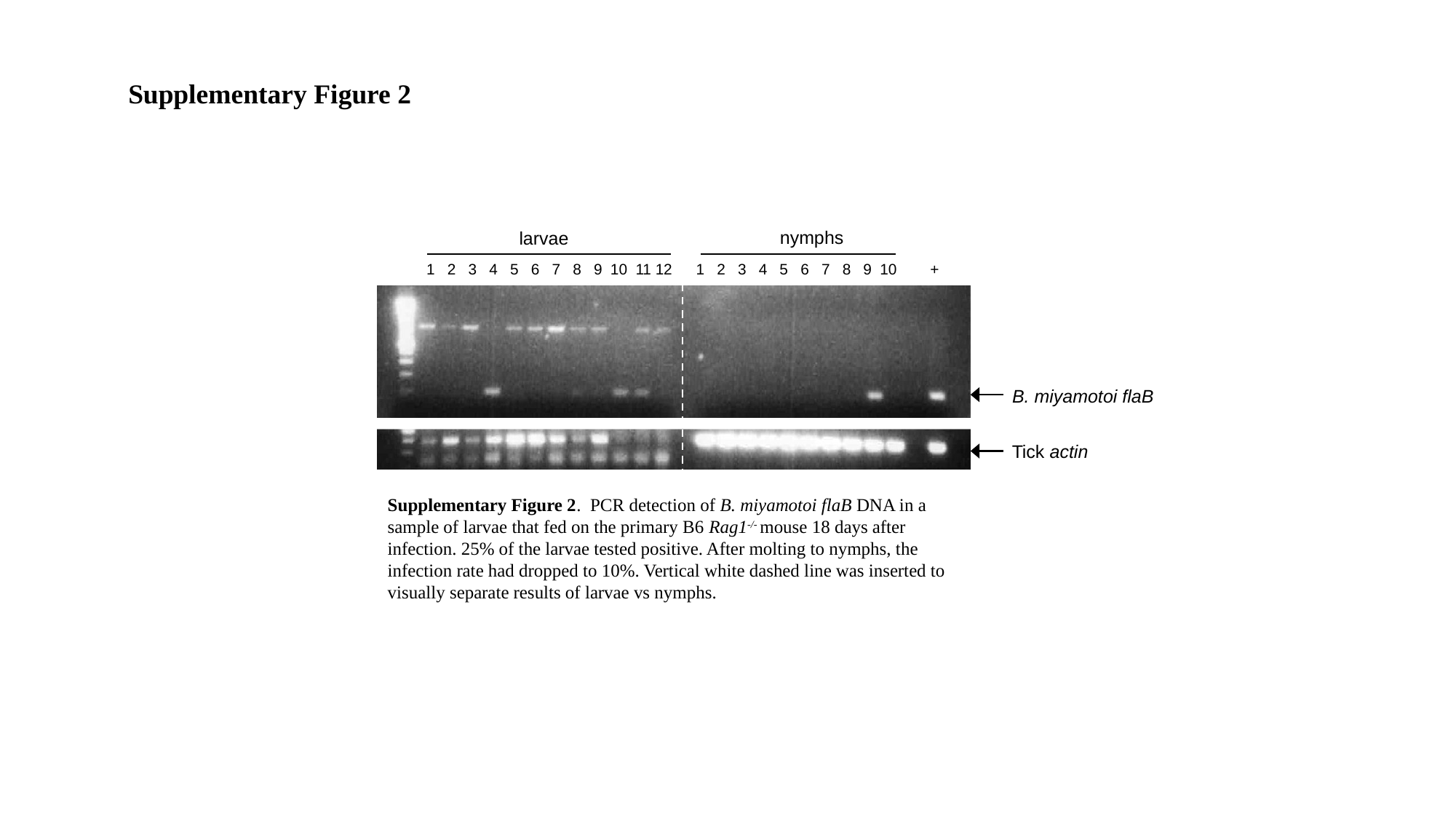

Supplementary Figure 2
nymphs
larvae
 1 2 3 4 5 6 7 8 9 10 11 12
 1 2 3 4 5 6 7 8 9 10 +
B. miyamotoi flaB
Tick actin
Supplementary Figure 2. PCR detection of B. miyamotoi flaB DNA in a sample of larvae that fed on the primary B6 Rag1-/- mouse 18 days after infection. 25% of the larvae tested positive. After molting to nymphs, the infection rate had dropped to 10%. Vertical white dashed line was inserted to visually separate results of larvae vs nymphs.

## Slide 4
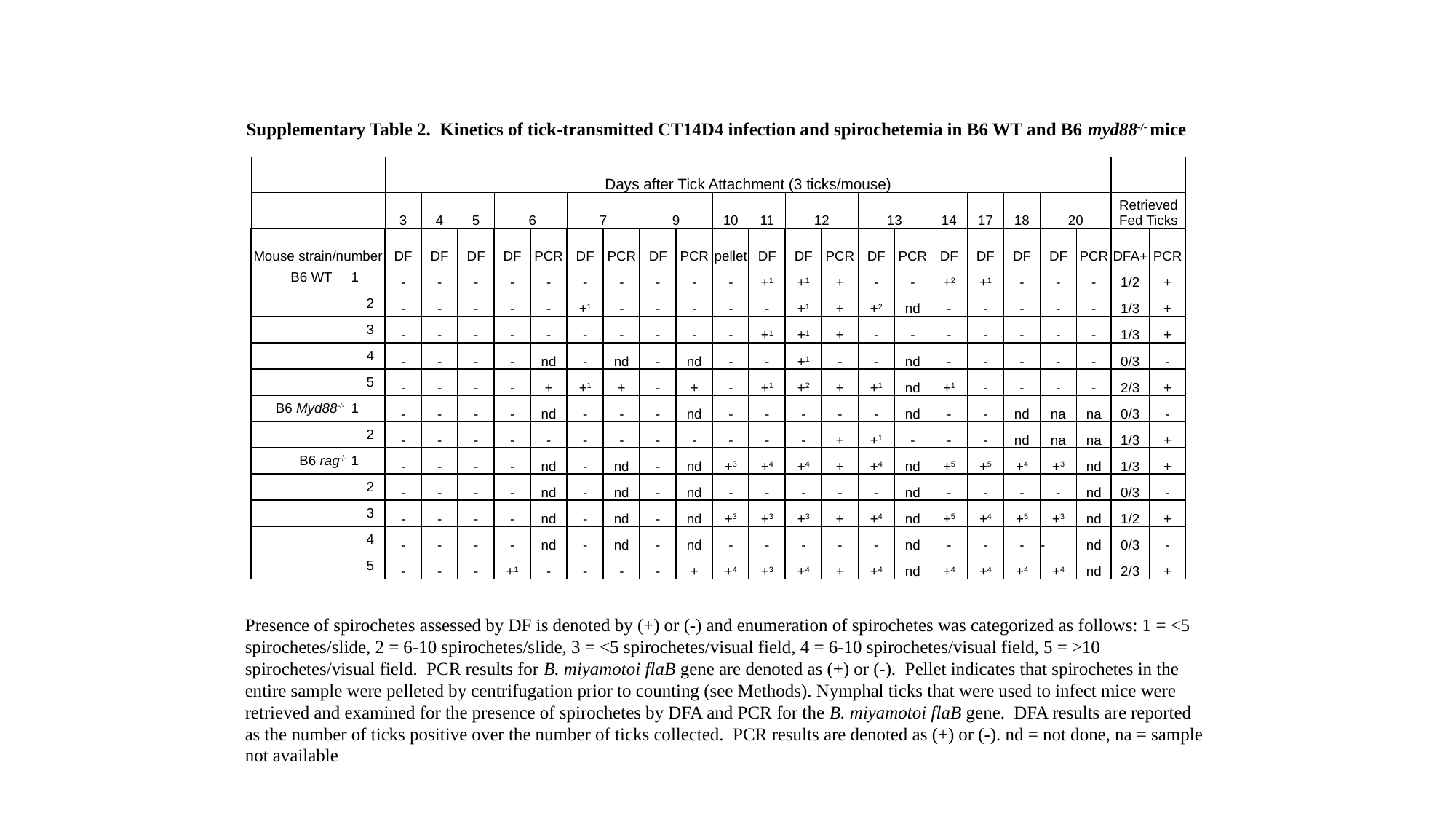

Supplementary Table 2. Kinetics of tick-transmitted CT14D4 infection and spirochetemia in B6 WT and B6 myd88-/- mice
| | Days after Tick Attachment (3 ticks/mouse) | | | | | | | | | | | | | | | | | | | | | |
| --- | --- | --- | --- | --- | --- | --- | --- | --- | --- | --- | --- | --- | --- | --- | --- | --- | --- | --- | --- | --- | --- | --- |
| | 3 | 4 | 5 | 6 | | 7 | | 9 | | 10 | 11 | 12 | | 13 | | 14 | 17 | 18 | 20 | | Retrieved Fed Ticks | |
| Mouse strain/number | DF | DF | DF | DF | PCR | DF | PCR | DF | PCR | pellet | DF | DF | PCR | DF | PCR | DF | DF | DF | DF | PCR | DFA+ | PCR |
| B6 WT 1 | - | - | - | - | - | - | - | - | - | - | +1 | +1 | + | - | - | +2 | +1 | - | - | - | 1/2 | + |
| 2 | - | - | - | - | - | +1 | - | - | - | - | - | +1 | + | +2 | nd | - | - | - | - | - | 1/3 | + |
| 3 | - | - | - | - | - | - | - | - | - | - | +1 | +1 | + | - | - | - | - | - | - | - | 1/3 | + |
| 4 | - | - | - | - | nd | - | nd | - | nd | - | - | +1 | - | - | nd | - | - | - | - | - | 0/3 | - |
| 5 | - | - | - | - | + | +1 | + | - | + | - | +1 | +2 | + | +1 | nd | +1 | - | - | - | - | 2/3 | + |
| B6 Myd88-/- 1 | - | - | - | - | nd | - | - | - | nd | - | - | - | - | - | nd | - | - | nd | na | na | 0/3 | - |
| 2 | - | - | - | - | - | - | - | - | - | - | - | - | + | +1 | - | - | - | nd | na | na | 1/3 | + |
| B6 rag-/- 1 | - | - | - | - | nd | - | nd | - | nd | +3 | +4 | +4 | + | +4 | nd | +5 | +5 | +4 | +3 | nd | 1/3 | + |
| 2 | - | - | - | - | nd | - | nd | - | nd | - | - | - | - | - | nd | - | - | - | - | nd | 0/3 | - |
| 3 | - | - | - | - | nd | - | nd | - | nd | +3 | +3 | +3 | + | +4 | nd | +5 | +4 | +5 | +3 | nd | 1/2 | + |
| 4 | - | - | - | - | nd | - | nd | - | nd | - | - | - | - | - | nd | - | - | - | - | nd | 0/3 | - |
| 5 | - | - | - | +1 | - | - | - | - | + | +4 | +3 | +4 | + | +4 | nd | +4 | +4 | +4 | +4 | nd | 2/3 | + |
Presence of spirochetes assessed by DF is denoted by (+) or (-) and enumeration of spirochetes was categorized as follows: 1 = <5 spirochetes/slide, 2 = 6-10 spirochetes/slide, 3 = <5 spirochetes/visual field, 4 = 6-10 spirochetes/visual field, 5 = >10 spirochetes/visual field. PCR results for B. miyamotoi flaB gene are denoted as (+) or (-). Pellet indicates that spirochetes in the entire sample were pelleted by centrifugation prior to counting (see Methods). Nymphal ticks that were used to infect mice were retrieved and examined for the presence of spirochetes by DFA and PCR for the B. miyamotoi flaB gene. DFA results are reported as the number of ticks positive over the number of ticks collected. PCR results are denoted as (+) or (-). nd = not done, na = sample not available

## Slide 5
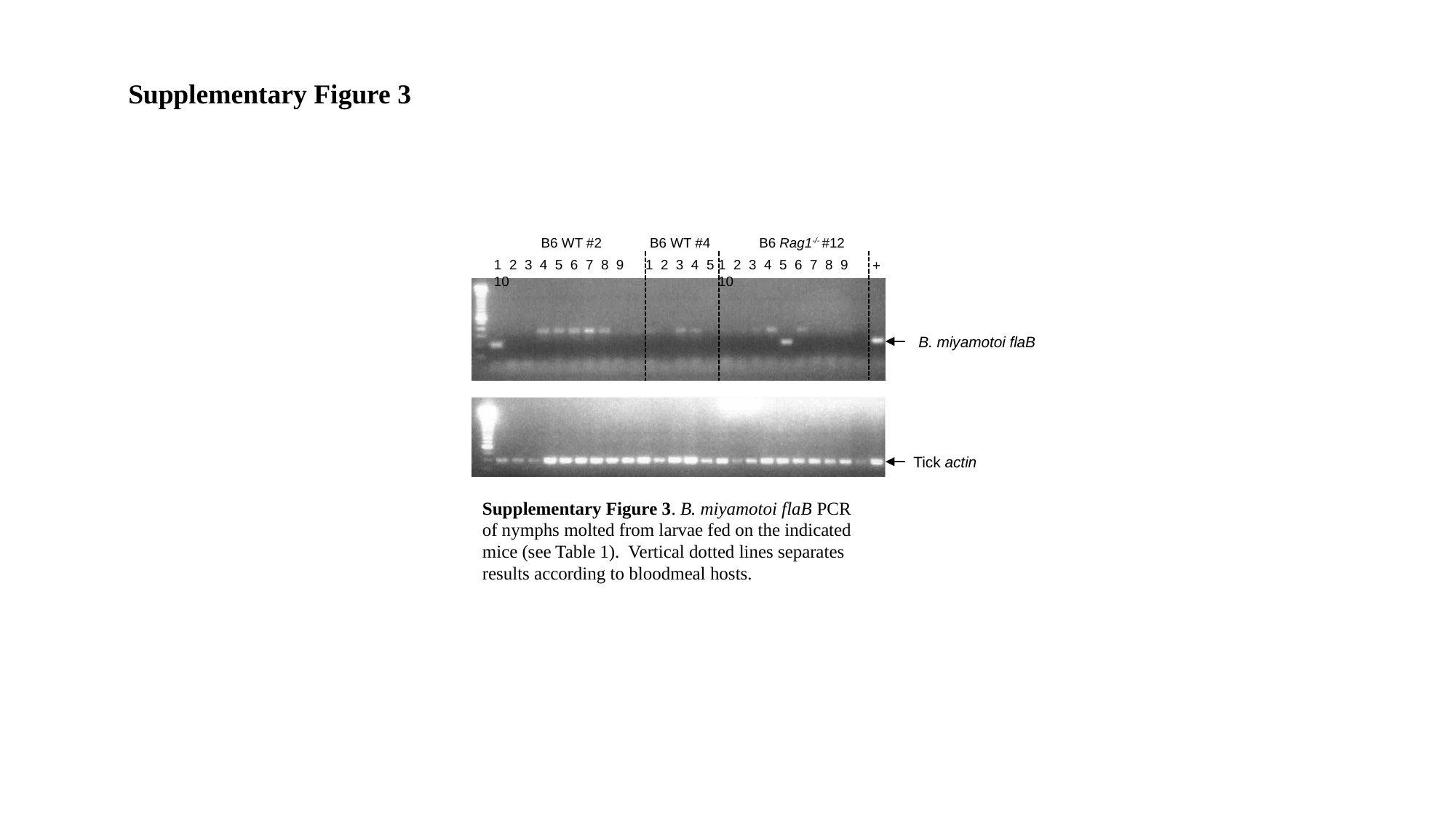

Supplementary Figure 3
B6 WT #2
B6 WT #4
B6 Rag1-/- #12
1 2 3 4 5 6 7 8 9 10
1 2 3 4 5
1 2 3 4 5 6 7 8 9 10
+
B. miyamotoi flaB
Tick actin
Supplementary Figure 3. B. miyamotoi flaB PCR of nymphs molted from larvae fed on the indicated mice (see Table 1). Vertical dotted lines separates results according to bloodmeal hosts.

## Slide 6
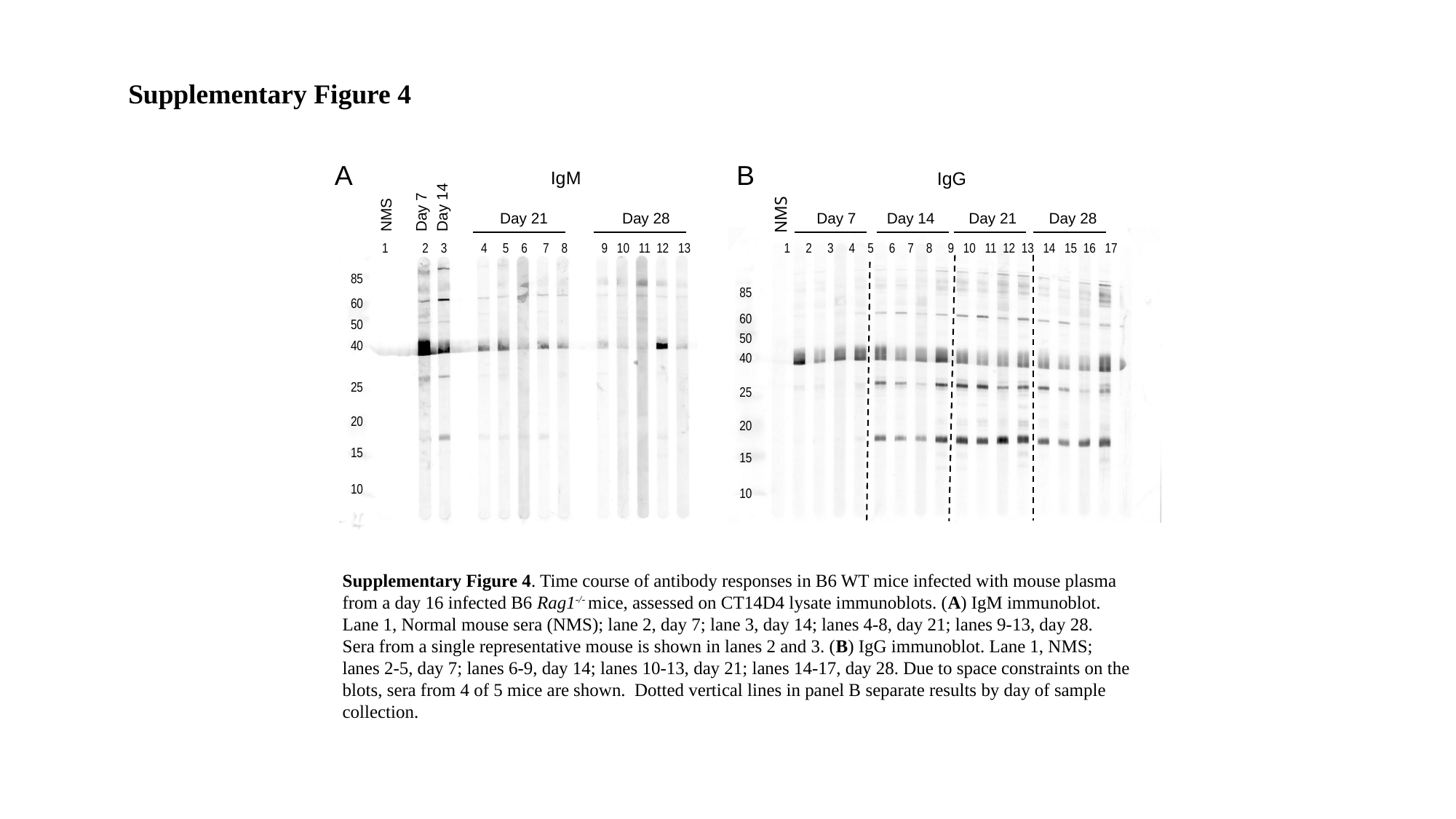

Supplementary Figure 4
B
A
IgM
IgG
Day 14
Day 7
NMS
NMS
Day 21
Day 28
Day 7
Day 14
Day 21
Day 28
1 2 3 4 5 6 7 8 9 10 11 12 13
1 2 3 4 5 6 7 8 9 10 11 12 13 14 15 16 17
85
60
50
40
25
20
15
10
85
60
50
40
25
20
15
10
Supplementary Figure 4. Time course of antibody responses in B6 WT mice infected with mouse plasma from a day 16 infected B6 Rag1-/- mice, assessed on CT14D4 lysate immunoblots. (A) IgM immunoblot. Lane 1, Normal mouse sera (NMS); lane 2, day 7; lane 3, day 14; lanes 4-8, day 21; lanes 9-13, day 28. Sera from a single representative mouse is shown in lanes 2 and 3. (B) IgG immunoblot. Lane 1, NMS; lanes 2-5, day 7; lanes 6-9, day 14; lanes 10-13, day 21; lanes 14-17, day 28. Due to space constraints on the blots, sera from 4 of 5 mice are shown. Dotted vertical lines in panel B separate results by day of sample collection.

## Slide 7
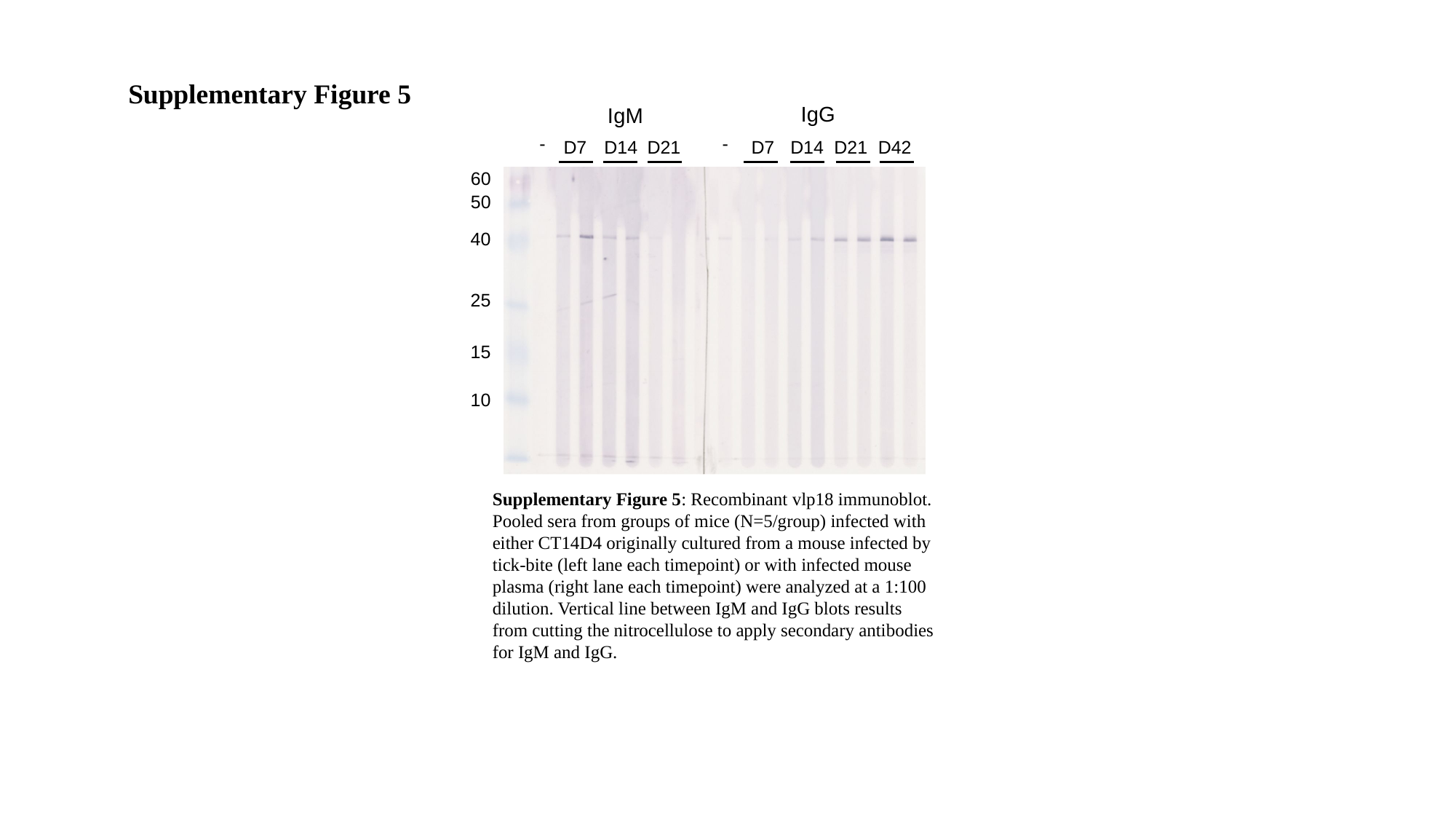

Supplementary Figure 5
IgG
IgM
-
-
D7
D14
D21
D7
D14
D21
D42
60
50
40
25
15
10
Supplementary Figure 5: Recombinant vlp18 immunoblot. Pooled sera from groups of mice (N=5/group) infected with either CT14D4 originally cultured from a mouse infected by tick-bite (left lane each timepoint) or with infected mouse plasma (right lane each timepoint) were analyzed at a 1:100 dilution. Vertical line between IgM and IgG blots results from cutting the nitrocellulose to apply secondary antibodies for IgM and IgG.

## Slide 8
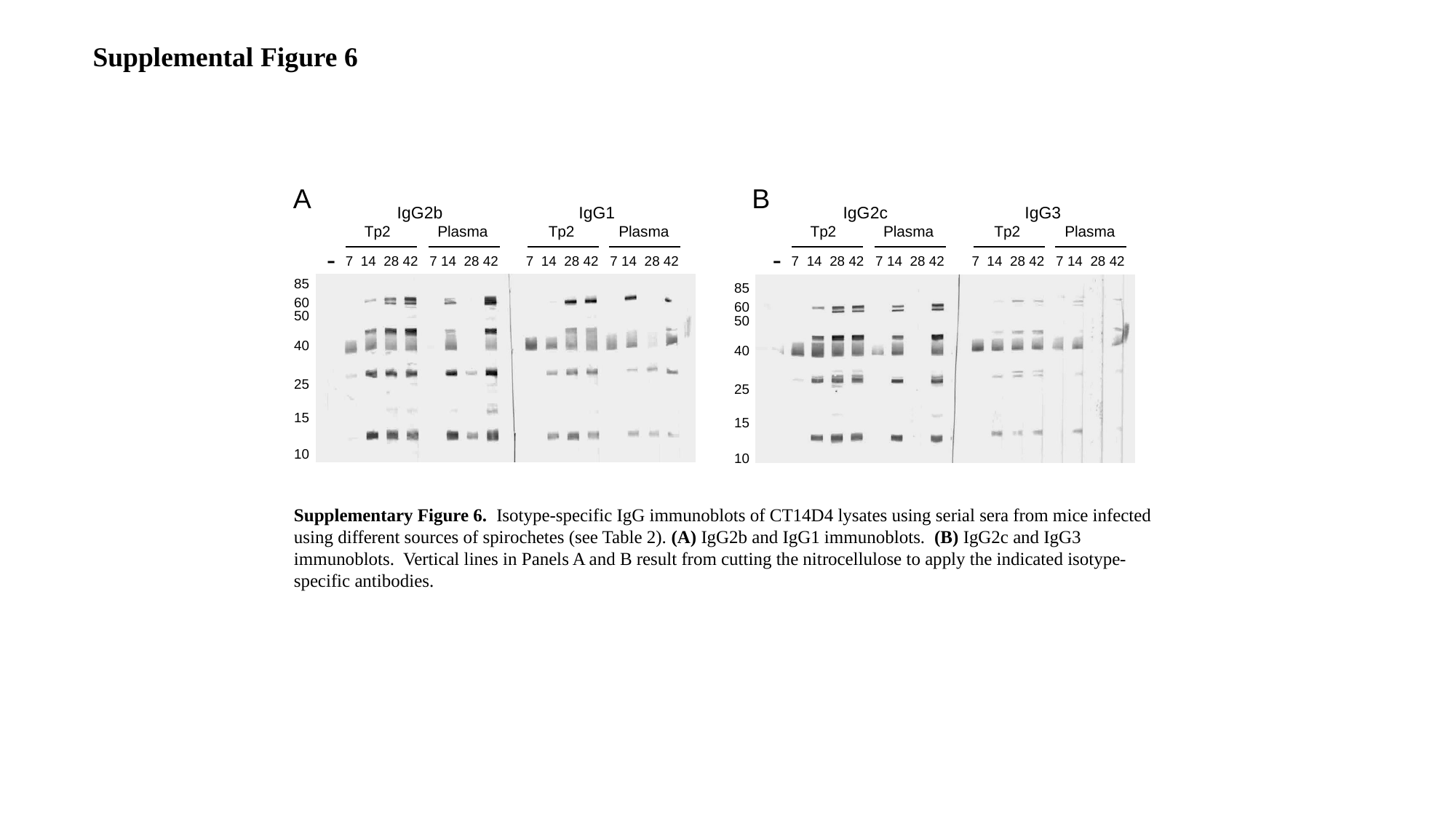

Supplemental Figure 6
A
B
IgG2b
IgG1
Tp2
Plasma
Tp2
Plasma
-
7 14 28 42 7 14 28 42
7 14 28 42 7 14 28 42
85
60
50
40
25
15
10
IgG2c
IgG3
Tp2
Plasma
Tp2
Plasma
-
7 14 28 42 7 14 28 42
7 14 28 42 7 14 28 42
85
60
50
40
25
15
10
Supplementary Figure 6. Isotype-specific IgG immunoblots of CT14D4 lysates using serial sera from mice infected using different sources of spirochetes (see Table 2). (A) IgG2b and IgG1 immunoblots. (B) IgG2c and IgG3 immunoblots. Vertical lines in Panels A and B result from cutting the nitrocellulose to apply the indicated isotype-specific antibodies.

## Slide 9
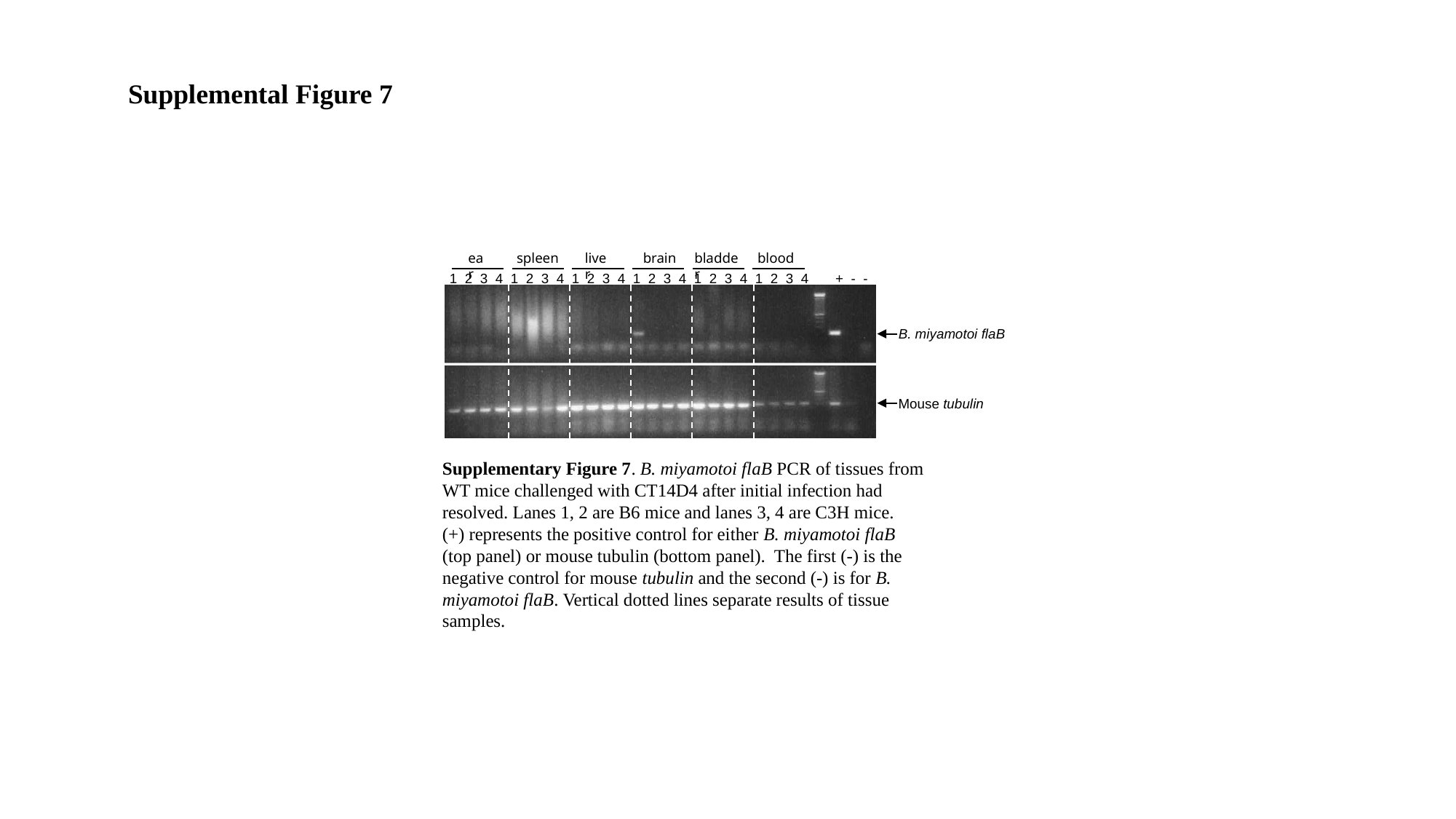

Supplemental Figure 7
ear
spleen
liver
brain
bladder
blood
1 2 3 4 1 2 3 4 1 2 3 4 1 2 3 4 1 2 3 4 1 2 3 4 + - -
B. miyamotoi flaB
Mouse tubulin
Supplementary Figure 7. B. miyamotoi flaB PCR of tissues from WT mice challenged with CT14D4 after initial infection had resolved. Lanes 1, 2 are B6 mice and lanes 3, 4 are C3H mice. (+) represents the positive control for either B. miyamotoi flaB (top panel) or mouse tubulin (bottom panel). The first (-) is the negative control for mouse tubulin and the second (-) is for B. miyamotoi flaB. Vertical dotted lines separate results of tissue samples.

## Slide 10
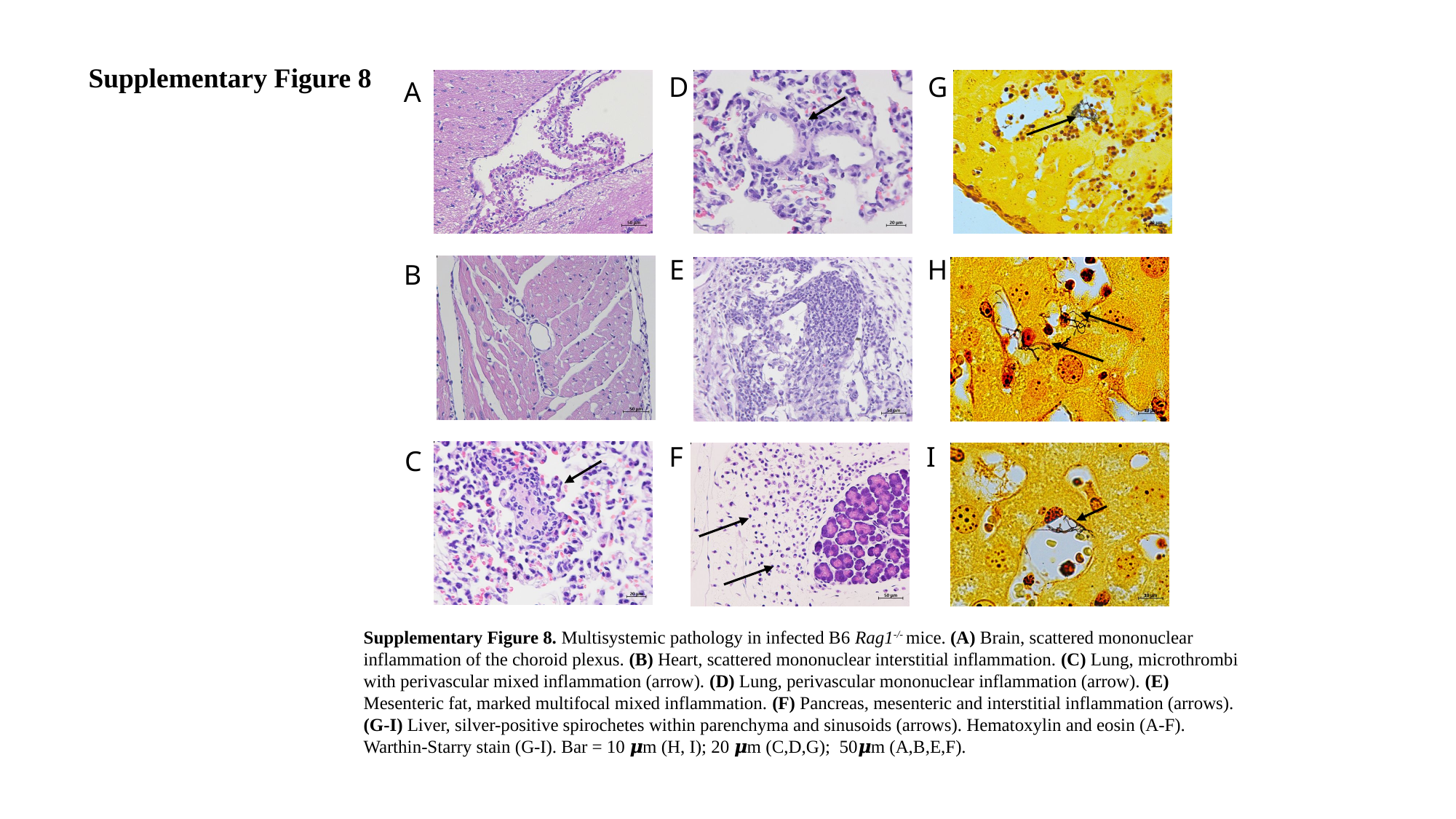

Supplementary Figure 8
D
G
A
E
H
B
F
I
C
Supplementary Figure 8. Multisystemic pathology in infected B6 Rag1-/- mice. (A) Brain, scattered mononuclear inflammation of the choroid plexus. (B) Heart, scattered mononuclear interstitial inflammation. (C) Lung, microthrombi with perivascular mixed inflammation (arrow). (D) Lung, perivascular mononuclear inflammation (arrow). (E) Mesenteric fat, marked multifocal mixed inflammation. (F) Pancreas, mesenteric and interstitial inflammation (arrows). (G-I) Liver, silver-positive spirochetes within parenchyma and sinusoids (arrows). Hematoxylin and eosin (A-F). Warthin-Starry stain (G-I). Bar = 10 𝝁m (H, I); 20 𝝁m (C,D,G); 50𝝁m (A,B,E,F).

## Slide 11
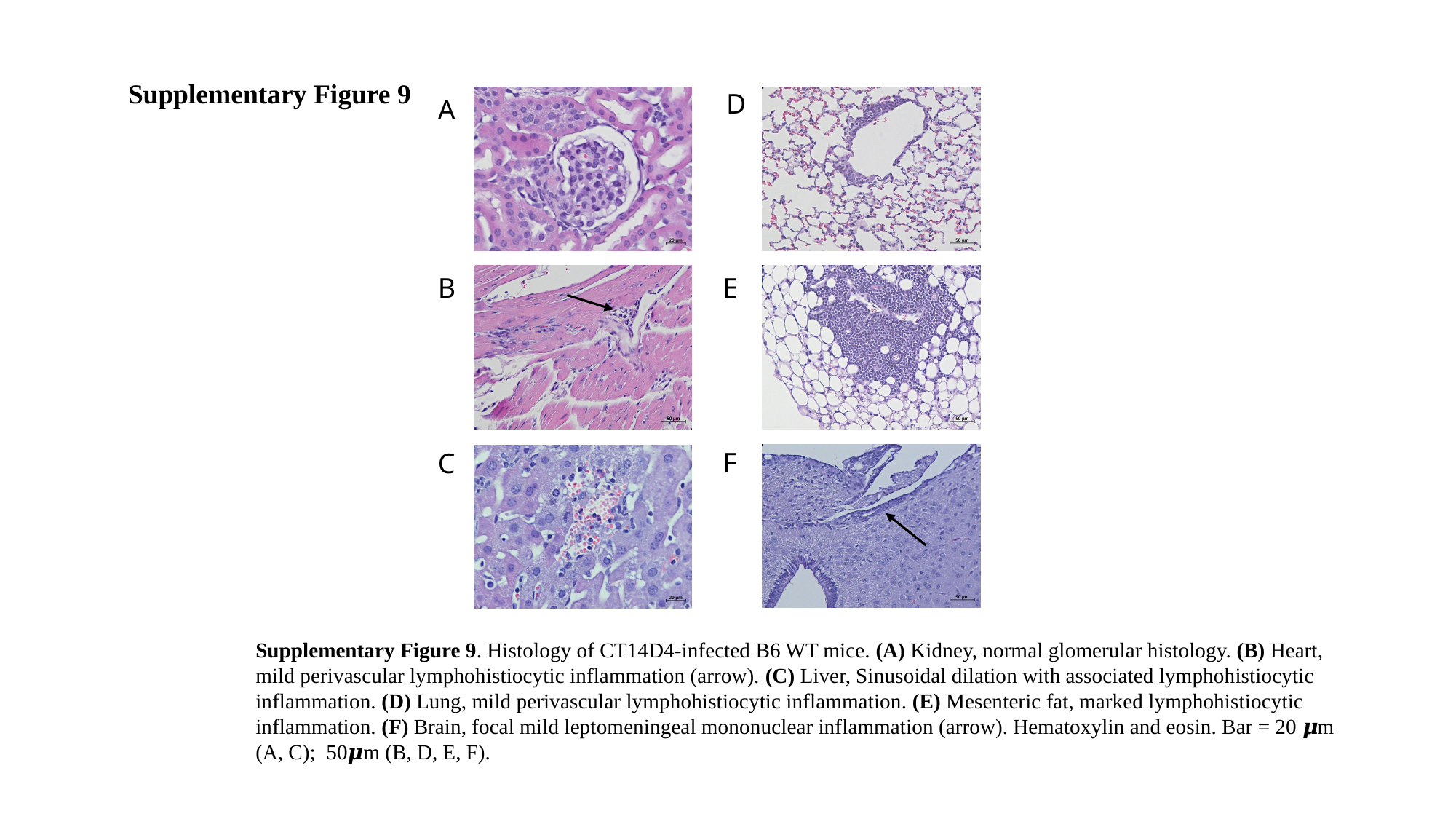

Supplementary Figure 9
D
A
B
E
F
C
Supplementary Figure 9. Histology of CT14D4-infected B6 WT mice. (A) Kidney, normal glomerular histology. (B) Heart, mild perivascular lymphohistiocytic inflammation (arrow). (C) Liver, Sinusoidal dilation with associated lymphohistiocytic inflammation. (D) Lung, mild perivascular lymphohistiocytic inflammation. (E) Mesenteric fat, marked lymphohistiocytic inflammation. (F) Brain, focal mild leptomeningeal mononuclear inflammation (arrow). Hematoxylin and eosin. Bar = 20 𝝁m (A, C); 50𝝁m (B, D, E, F).

## Slide 12
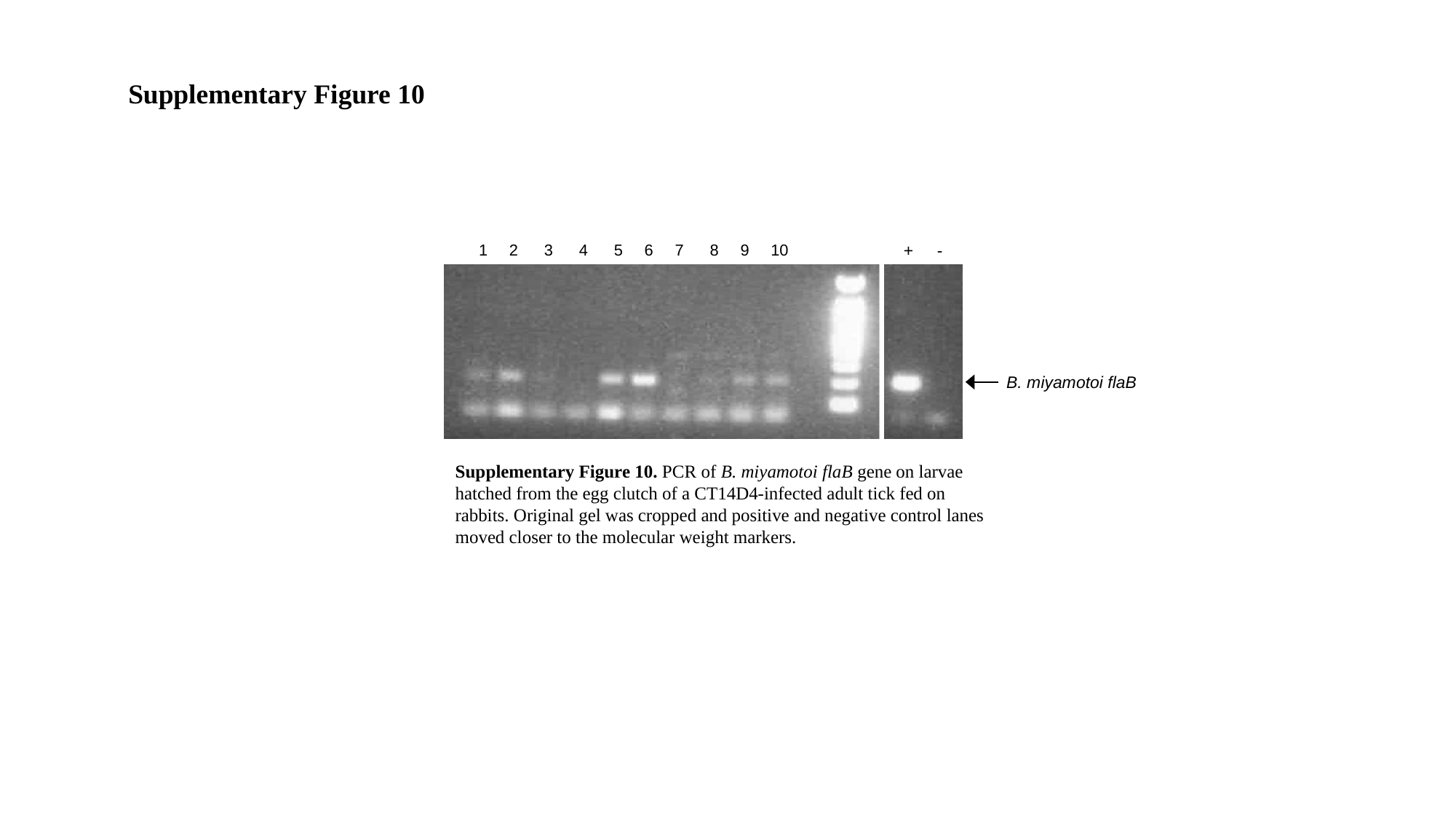

Supplementary Figure 10
1 2 3 4 5 6 7 8 9 10
+ -
B. miyamotoi flaB
Supplementary Figure 10. PCR of B. miyamotoi flaB gene on larvae hatched from the egg clutch of a CT14D4-infected adult tick fed on rabbits. Original gel was cropped and positive and negative control lanes moved closer to the molecular weight markers.
